# Single-cell transcriptomic analysis of endometriosis provides insights into fibroblast fates and immune cell heterogeneity

**DOI:** 10.1186/s13578-021-00637-x

**Published:** 2021-07-07

**Authors:** Junyan Ma, Liqi Zhang, Hong Zhan, Yun Mo, Zuanjie Ren, Anwen Shao, Jun Lin

**Affiliations:** 1 Key Laboratory of Women’s Reproductive Health Research of Zhejiang Province, Women’s Hospital, Zhejiang University School of Medicine, Hangzhou, China; 2 Department of Obstetrics and Gynecology, Women’s Hospital, School of Medicine, Zhejiang University, Hangzhou, China; 3 Department of Neurosurgery, The Second Affiliated Hospital, Zhejiang University School of Medicine, Hangzhou, China

**Keywords:** Endometriosis, Single-cell sequence, Fibroblast, Immune cell heterogeneity

## Abstract

**Background:**

Endometriosis is an oestrogen-dependent disease with an unclear aetiology and pathogenesis affecting 6–10% of the global female population, predominantly those of reproductive age. Herein, we profile the transcriptomes of approximately 55,000 single cells from three groups including ectopic endometrium, eutopic endometrium from women with endometriosis, and eutopic endometrium from healthy women to create a single-cell transcriptome atlas of endometriosis.

**Results:**

We have identified 9 cell types and performed single-cell analysis of fibroblasts, and determined a potential developmental trajectory associated with endometriosis. We also identified fibroblast subpopulations related to endometriosis development and found that *StAR* played an important role in this process. Moreover, T cells in endometriosis were less activated or inflammatory with decreased effector CD8 + T cells, while the composition ratio of natural killer cells decreased and the percentage of monocytes/macrophages increased in endometriosis cysts. In addition, the effectiveness of immune cells in endometriosis lesions, eutopic endometrium from women with endometriosis, and eutopic endometrium from healthy women was distinct. Cell–cell interaction analyses highlighted the imbalanced immune environment in endometriosis lesions and immune cells in endometriosis could promote the development of the disease.

**Conclusion:**

Our study provided a systematic characterisation of endometriosis and insights into the aetiology and pathology of endometriosis.

**Supplementary Information:**

The online version contains supplementary material available at 10.1186/s13578-021-00637-x.

## Background

Endometriosis, a common, benign, gynaecological disease associated to pelvic pain and infertility [1, 2], is characterised by ectopic endometrial-like tissues that are often altered by menstrual cycle hormones. This disease affects 6–10% of the global female population, predominantly those of reproductive age [1, 3–5]. Moreover, its highly variable symptoms make diagnosis challenging, often resulting in misdiagnosis. The current gold standard for diagnosis is invasive laparoscopy or laparotomy, while the disease severity can be assessed using a scoring system [6]. Currently, no effective curative measures are available, primarily due to its unclear aetiology and pathogenesis. Although the dystonia retrograde menstruation theory is the most widely accepted, yet commonly disputed, theory explaining endometriosis aetiology [7–9], no current theories fully define the pathogenesis of endometriosis.

Accordingly, recent studies have attempted to elucidate the molecular and cellular mechanisms underlying endometriosis pathogenesis [9–13]; yet these studies have reported conflicting conclusions when comparing endometriosis and the eutopic endometrium to healthy endometrial tissue, as each cell population has a distinct transcriptome and integrative canonical correlation analysis tends to mask heterogeneity within individual cell populations. Alternatively, single-cell RNA-sequencing (scRNA-seq) is suitable for characterising this heterogeneity, facilitating identification of closely related cell populations based on expression of established markers [14]. Hence, scRNA-seq can assess heterogenous gene expression during health and disease states, and may, therefore, highlight relevant disease processes [15, 16].

Herein, we applied scRNA-seq to comprehensively classify the transcriptional profiles of nine cell types, and compared endometriosis, eutopic endometrium, and normal endometrium in each cell population and identified cell subtypes implicated in endometriosis.

## Results

### Study population

**Fig. 1:**
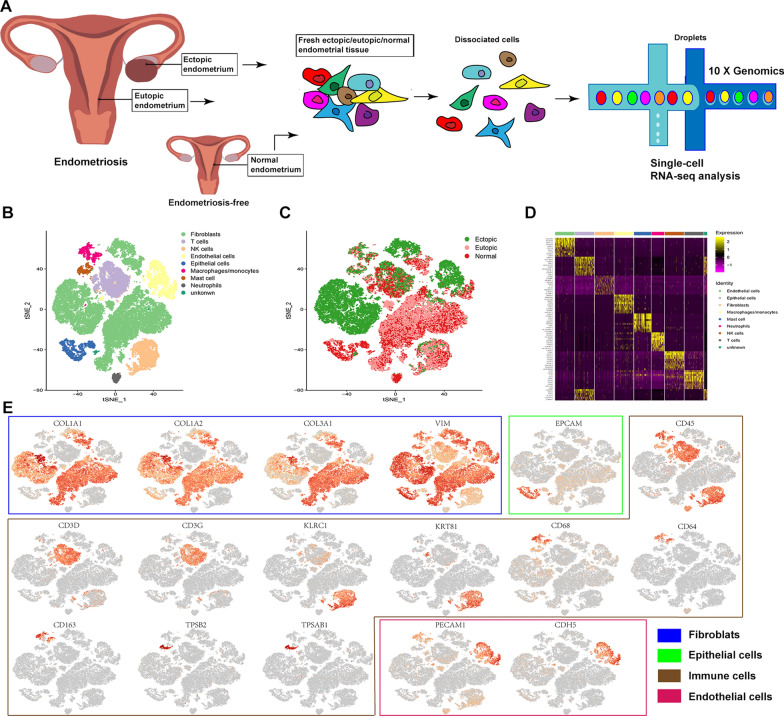
Integrated single-cell RNA-seq analysis of patients with endometriosis and without the disease identifies diverse cell populations. Single-cell RNA-seq was performed on single-cell suspensions generated from three ovarian endometriosis samples and three eutopic endometrium samples from the same patient and three normal endometria from patients without the disease. All nine samples were analysed using canonical correlation analysis within the Seurat R package (version 3.0). Cells were clustered using a graph-based shared nearest neighbour clustering approach and visualised using a t-distributed stochastic neighbour embedding (t-SNE) plot. **A** Workflow diagram showing the collection and processing of fresh samples from three endometriosis patients and three patients without the disease for scRNA-seq. **B** t-SNE plots of cells from the nine samples profiled in this study, with each cell colour coded to indicate the associated cell types. **C** Cells on the t-SNE plot of all nine samples were coloured as originating either from patients with endometriosis or from patients without endometriosis. **D** Expression phenotypes of markers in these clusters are shown in the heat map (each column represents an individual cluster; pink represents the minimum, black represents the median, and yellow represents the maximum expression values). These subpopulations were hierarchically clustered based on their marker expression patterns. **E** Canonical cell markers were used to label clusters by cell identity as represented in the t-SNE plot. Cell types were classified as fibroblasts (FBs), epithelial cells (Eps), endothelial cells (ECs), or immune cells as indicated in the legend

We acquired single-cell transcriptome profiles (10 × Genomics Chromium system) from nine clinical samples and divided them into three groups, including three ectopic endometrium from endometriosis lesion, three eutopic endometrium from women with endometriosis, and three normal endometrium from women without endometriosis (Fig. 1A; Additional file 1: Fig. S1; Table 1). A total of 67,103 single cells were obtained, of which 55,188 passed stringent quality control filters for subsequent analysis. All data reached a median depth of 53,111–102,997 reads/cell and 1762–3199 genes/cell (Additional file 2). To acquire comprehensive observations of the cellular composition, we used a graph-based clustering approach and t-distributed stochastic neighbour embedding (t-SNE) with Seurat to classify principal cell clusters.

**Table 1:** Characteristics of patients included in this study

Patient characteristics							
Patient	Diagnosis	Age	BMI	Menstruation	Menstrual cycle	Disease stage	Clinical symptoms
Patient 1	Ovarian endometriosis	33	22.9	Regular	Proliferation	III	Dysmenorrhea
Patient 2	Ovarian endometriosis	29	19.1	Regular	Proliferation	III	Dysmenorrhea
Patient 3	Ovarian endometriosis	33	20.9	Regular	Proliferation	IV	Infertility
Mean ± SD		**31.66666667**	**22.4 ± 3.3 (NS)**	Regular	Proliferation		
Patient 4	Without endometriosis	26	23.6	Regular	Proliferation		No obvious symptoms
Patient 5	Without endometriosis	34	25	Regular	Proliferation		No obvious symptoms
Patient 6	Without endometriosis	26	17.1	Regular	Proliferation		No obvious symptoms
Mean ± SD		**30.38095238**	**24.2 ± 4.0 (NS)**	Regular	Proliferation		

*BMI*  body mass index, *SD* standard deviation, *NS* no significant

### ScRNA-seq analysis reveals multiple cell populations in ovary endometriosis, eutopic endometrium and normal endometrium

ScRNA-Seq categorised the entire cell population into nine major cell clusters, from which cell types, including fibroblasts (FBs), endothelial cells (ECs), epithelial cells (Eps), T cells, natural killer cells (NK), macrophages/monocytes (M), mast cells, and neutrophils, were identified based on expression of markers identified by SingleR (Fig. 1B–E). The complete table of differentially expressed genes (DEGs) is shown in Additional file 3. Subsequently, Gene Ontology (GO) enrichment analysis and Kyoto Encyclopedia of Genes and Genomes (KEGG) pathway analysis with up-expressed genes identified specific processes relevant to them (Additional file 1: Fig. S2), which were coincident with their cell type.

### ScRNA-seq analysis reveals characteristic differences and similarities between cell subgroups

**Fig. 2:**
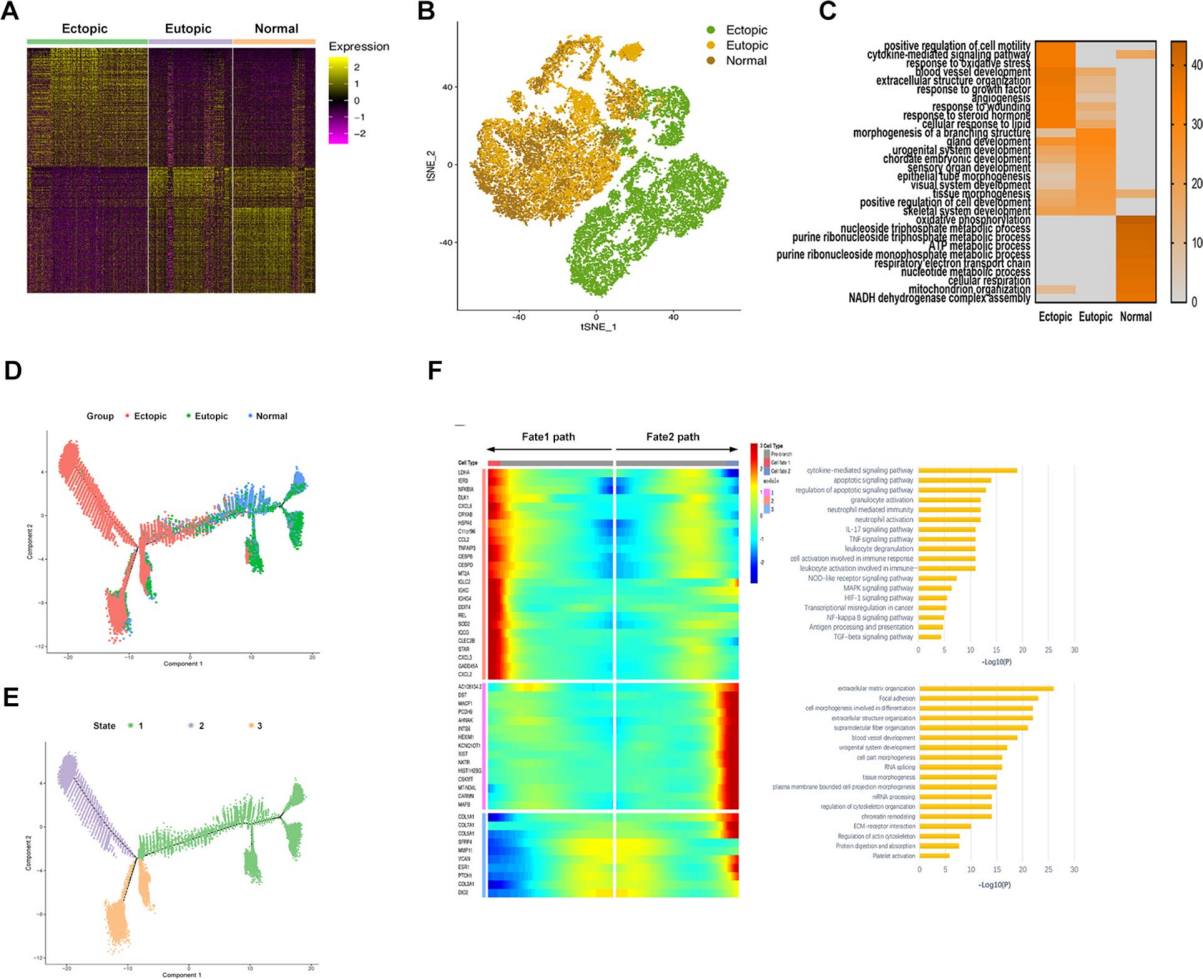
Subclusters and pseudotime trajectory of FBs in endometriosis lesions, eutopic endometrium and normal endometrium. **A** Differential expression analysis of FBs genes was performed comparing FBs from endometriosis lesions, eutopic endometrium and normal endometrium. Heatmaps are shown representing upregulated and downregulated genes. The full table of genes is shown in Additional file 11. **B** FBs on the t-SNE plot of all nine samples are coloured as originating either from patients with endometriosis or without endometriosis. **C** Functional enrichment analysis with GO and KEGG was performed with significantly upregulated genes in each group. Representative significantly enriched function processes are shown. **D**, **E** Monocle analyses showing the development of FBs along the pseudotime trajectory. **F** Heatmap of different blocks of DEGs along the pseudotime trajectory. The full table of DEGs is shown in Additional file 5. Functional enrichment analysis with GO and KEGG analysis was performed with the enriched genes in cells from fates 1 and 2

Using similarity analyses, we found numerous distinctions in the composition ratio and structure of the cell types, as well as in the expression of genes within the clusters, suggesting that the biological features of endometriosis lesions differed from those of eutopic endometrium. Furthermore, although the eutopic endometrium from endometriosis patients resembled normal endometrium, differences in both cell composition and gene expression, particularly when comparing cell subclusters, were observed (Figs. 2A, B, 4B–D; Additional file 1: Fig. S3A, B, Fig. S4). Furthermore, GO and KEGG analyses with up-expressed genes identified different enriched biological processes among three groups, suggesting that endometriosis was associated with angiogenesis, ECM organisation, cell motility, adhesion, and response to wounding. Meanwhile, normal endometrium was associated with metabolic processes, and eutopic endometrium presented a transitional state of endometriosis and normal endometrium (Additional file 1: Fig. S3C). The sc-RNA-seq data quantified expression of genes associated with disease developmental pathways, including TGF-β, MAPK, Rho-Rack, NF-κB, and JAK-STAT, as well as signalling pathways associated with epithelial-to-mesenchymal transition (EMT) [17–19], in various endometrial cell populations (Additional file 1: Fig. S3D). In endometriosis lesions, we observed upregulation of ligands and target gene expression of these pathways in epithelial cells, with few changes in immune cells. We also detected downregulated expression of several MAPK pathway genes in endothelial cell populations. Several genes related to EMT, NF-κB, and JAK-STAT pathways were upregulated in FBs within endometriosis lesions.

### Fibroblasts are important etiological factors in endometriosis

#### ScRNA-seq revealed different enrichment function of FBs in endometriosis lesions, eutopic endometrium and normal endometrium

As the main cell component in the disease lesion, FBs within endometriosis lesions, eutopic endometrium, and normal endometrium were compared and various DEGs were identified (Fig. 2A, B; Additional file 4). Moreover, GO and KEGG enrichment analyses revealed that within endometriosis lesions, FBs were enriched for genes associated with cell motility, extracellular structure organisation, angiogenesis, and cytokine-mediated signalling pathways. Eutopic FBs were enriched with functions including gland development and tissue morphogenesis, while normal endometrium FBs were related to ATP and metabolic processes (Fig. 2C). Interestingly, the results showed a process of gradual change in function from normal endometrium to eutopic endometrium, and finally to ectopic endometrium. To further clarify the pathogenic genes associated with disease-related FBs, we analysed our scRNA-seq data to explore DEGs that highly expressed in endometriosis FBs, including *C3*, *C7*, *StAR* and *S100A10*. *C3* and *C7* were important components in the complement system which participated in inflammation as well as supported tumour growth [20]. *StAR* was essential for catalytic conversion of cholesterol to E2, which was necessary for endometriosis development [21]. *S100A10* could activate the plasminogen activation pathway, increase levels of plasmin to promote degradation of the ECM and increase angiogenesis and invasion properties [22]. Immunofluorescence confirmed that *C3*, *C7*, *StAR* and *S100A10* were expressed more abundantly in endometriosis FBs (Additional file 1: Fig. S5).

#### Mapping the developmental track of FBs by pseudotime state transition

To further comprehend the role of FBs in endometriosis, we applied pseudotime methods to investigate the differentiation trajectory of FB populations. This unsupervised approach identified a continuum of cell states and showed two distinct trajectories beginning at state 1 and gradually progressing toward states 2 and 3, revealing a common origin with divergent fates. State 1 comprised primarily FBs from eutopic and normal endometrium, which then segregated into two branches in pseudotime (Fig. 2D–E). While one branch described the developmental pathway of FBs predominantly from endometriosis lesions and euctopic endometrium (fate 1), the other was occupied by FBs derived from endometriosis cyst and endometrium from women with and without disease (fate 2) (Additional file 1: Fig. S6A). Notably, the trajectory underpinning the ectopic endometrium and euctopic endometrium significantly overlapped, while the euctopic FBs tended to differentiate into ectopic FBs in the fate 1 trajectory. Furthermore, we identified genes differentially expressed by states that were progressively altered in the two branches versus the pre-branch (Fig. 2F; Additional file 5). We found that the cells undergoing fate 1 expressed appreciably higher levels of genes previously defined as pathogenic compared to cells that underwent fate 2, such as *CCL2, CXCL2, CXCL3*, and *StAR*, suggesting that fate 1 represented a transition along a spectrum of pathogenic states. However, the fate 2 pathway was enriched with cells expressing homeostatic molecules, including COL3A1 and VCAN, which are associated with normal endometrium development.

GO and KEGG analyses showed that genes highly expressed in fate 1 were enriched in functions related to chemokine-mediated signalling pathways, immune response, and TNF and MAPK signalling pathways, while functions related to ECM-receptor interaction and tissue morphogenesis were expressed in fate 2 (Fig. 2F). These functional enrichments closely reflected the molecular features of FBs in the two routes and further explained our observations. Together, these findings demonstrated the complexity of FB development in endometriosis and indicated that the trajectory of fate 1 predisposed pathogenic development of endometriosis.

#### ScRNA-seq reveals important fibroblast subpopulations related to endometriosis

**Fig. 3:**
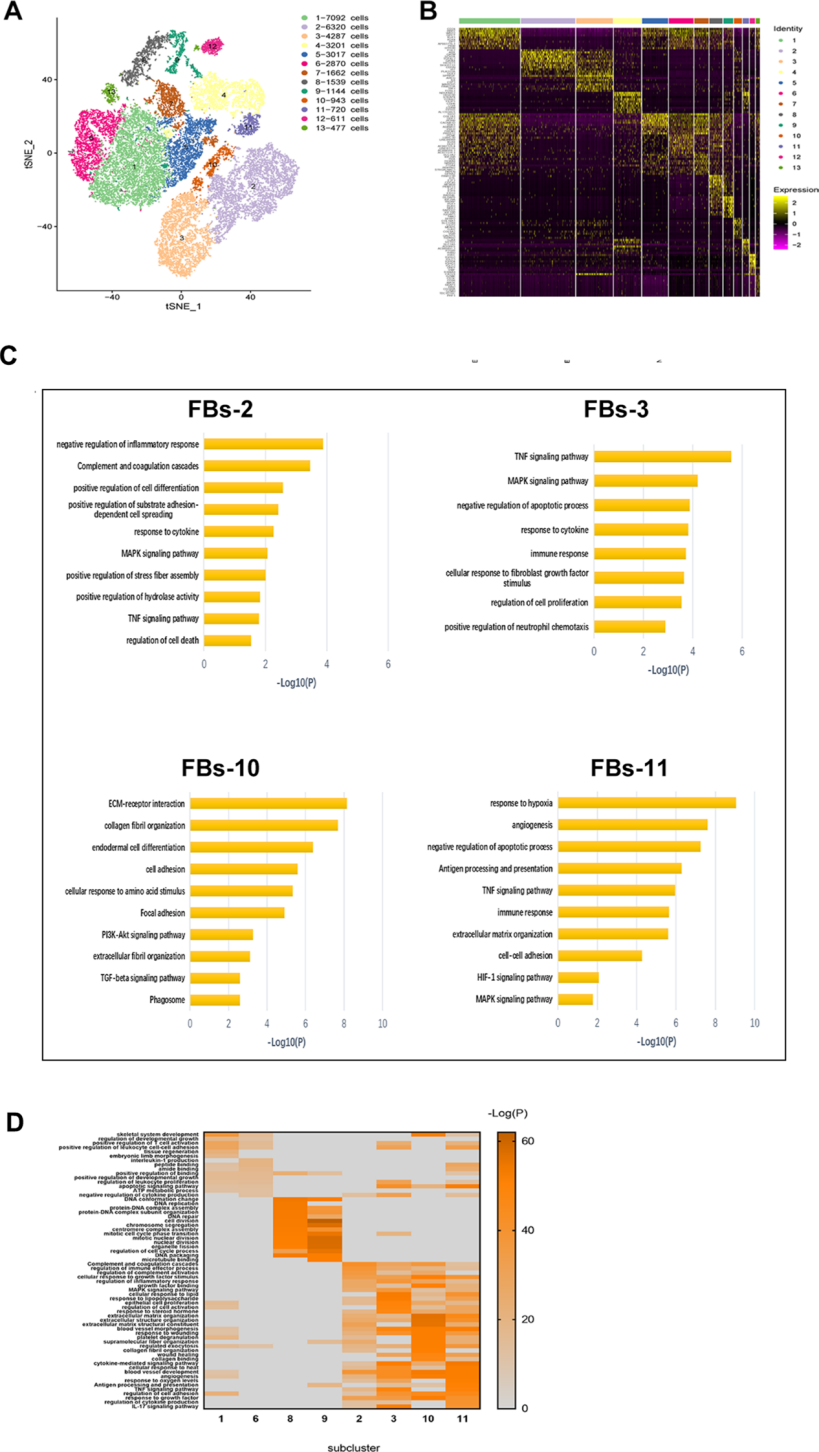
Distinct characteristics of FBs subpopulations. **A** Cells identified as FBs by individual annotation of scRNA-seq data from nine samples were combined and then clustered, revealing 13 clusters. **B** Expression phenotypes of markers in each subcluster of FBs are shown in the heat map. The full table of marker genes is presented in Additional file 4. **C** Functional enrichment analyses with GO and KEGG of FBs-2, FBs-3, FBs-10, and FBs-11. **D** Functional enrichment analyses with GO and KEGG were compared in characteristic subclusters from three groups

To identify the intrinsic structure and potential functional subtypes of the entire FBs population, we performed unsupervised clustering of all cells identified as FBs to examine their heterogeneity. We identified a total of 13 stable clusters, each with unique signature genes (Fig. 3A, B), and observed a significant difference in subtype composition among ectopic endometrium, eutopic endometrium, and normal endometrium. Although the eutopic endometrium generally resembled the normal endometrium, differences were apparent when comparing the cell subtypes. Specifically, SC-FBs-2, SC-FBs-3, SC-FBs-10, and SC-FBs-11 appeared in endometriosis lesions, whereas SC-FBs-1, SC-FBs-6, SC-FBs-8, and SC-FBs-9 existed almost exclusively in the endometrium (Additional file 1: Fig. S6B, C). Closer inspection of endometriosis-associated FBs subclusters and their signature genes showed their functional characteristics. SC-FB-2 were associated with the cytokine and inflammatory response, SC-FBs-3 were relevant to fibroblast growth factor stimulus and immune response, SC-FBs-10 were associated with ECM and cell adhesion, and SC-FBs-11 were correlated with angiogenesis and hypoxia response. All four FB subclusters were involved in MAPK, TNF, IL-17, and TGF-β signalling pathways [23–25], which supported the disease state, and were relevant to the development of endometriosis (Fig. 3C).

To further elucidate the functional differences among FB subclusters, we compared enriched functions of DEGs using GO and KEGG analysis. SC-FBs-2, SC-FBs-3, SC-FBs-10, and SC-FBs-11 were enriched with functions including extracellular structure organisation, wound healing, cell adhesion, and the MAPK signaling pathway; whereas SC-FBs-1, SC-FBs-6, SC-FBs-8, and SC-FBs-9 were more related to physiological functions (Fig. 3D). These results defined the disease-related FB subgroups and provided evidence for the important role of FBs in endometriosis pathogenesis.

High expression of *StAR* in FBs played an important role in the development of endometriosis, and its effect was assessed in this study. We downregulated the expression of *StAR* with siRNA, which was confirmed using western blot analysis (Additional file 1: Fig. S6D). In comparison with si-Ctrl group, the abilities of migration and invasive were significantly reduced in si-*StAR* FBs, as evaluated using transwell and wound scratch assays (Additional file 1: Fig. S6E, F). Meanwhile, cell counting kit-8 assay (CCK8 assay) revealed an obvious decrease of proliferative ability in the si-*StAR* FBs compared to si-Ctrl groups (Additional file 1: Fig. S6G). Results from flow cytometric analysis also indicated that the percentage of G1 phase cells was higher in the si-*StAR* FBs than in si-Ctrl groups (Additional file 1: Fig. S6H). In addition, the apoptosis rate of FBs was increased following *StAR* downregulation (Additional file 1: Fig. S6I). These findings suggested that *StAR* overexpression in FBs might increase its ability of proliferation, migration and invasion.

### ScRNA-seq analysis reveals immune cells characteristics in endometriosis lesions, eutopic endometrium and normal endometrium

**Fig. 4:**
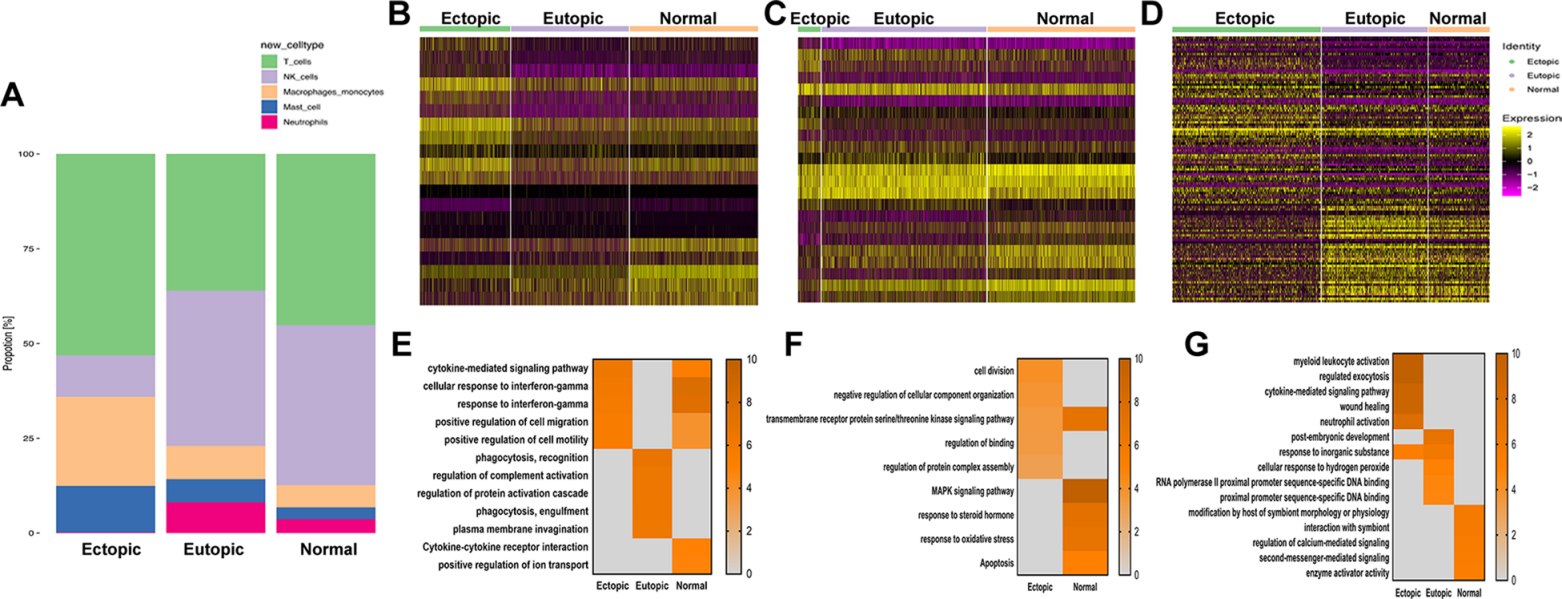
Immune cells clusters in endometriosis lesions, eutopic endometrium and normal endometrium. **A** The relative ratio of immune cells in endometriosis lesions, eutopic endometrium and normal endometrium shown using bar plots. **B**–**D** Differential expression analysis of genes was performed and shown using heatmaps in T cells, NK, and M cells. **E**–**G** Functional enrichment analysis of T cells, NK, and M cells with GO and KEGG analysis were compared among the three groups. Representative significantly enriched function processes are shown

Using our scRNA-seq data, we sought to further characterise the microenvironment of endometriosis cysts to anatomise the associated immune cells. Comparing the relative proportions, we found that immune cells differed significantly between normal endometrium and euctopic endometrium as well as ectopic endometrium and eutopic endometrium from those with endometriosis. While the ratio of NK cells was significantly decreased in the lesions (10.9% in endometriosis lesions vs 40.9% in eutopic endometrium and 42.2% in normal endometrium) and the percentage of M cells was higher (23.5% in endometriosis lesions vs 8.7% in eutopic endometrium and 5.8% in normal endometrium) (Fig. 4A; Additional file 6).

We then compared gene expression in T, NK, and M cells among the three groups (Fig. 4B–D), and identified unique functions enriched in all groups (Fig. 4E–G), which further clarified the differences in the immune microenvironment among endometriosis lesions. We then performed dimensionality reduction and clustering within these immune cells and observed multiple subclusters in each cell type with distinct molecular features. These subclusters displayed shifts in composition among endometriosis and endometrium from patients with or without endometriosis. We then performed further analysis on these cells as follows:

#### T cells

GO and KEGG analyses suggested that T cells in endometriosis were less activated. Moreover, the upregulated genes in eutopic endometrium were enriched in functions including phagocytosis and complement activation (Fig. 4E).

**Fig. 5:**
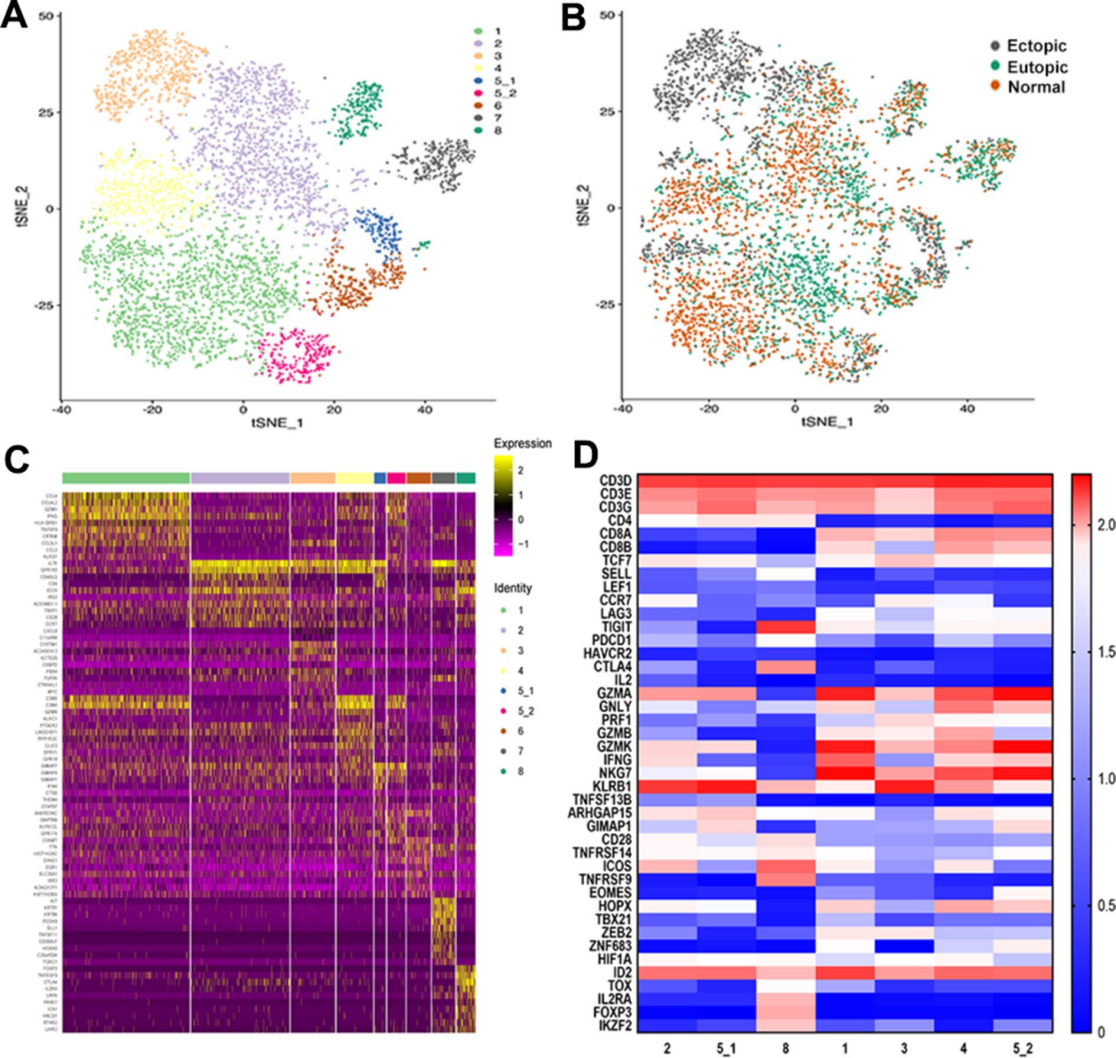
Distinct characteristics of T cells clusters in endometriosis lesions, eutopic endometrium and normal endometrium. **A** Seven subclusters were identified after T cells were combined and clustered. **B** T cells on the t-SNE plot of all nine samples are coloured as originating either from patients with endometriosis or without endometriosis. **C** Heatmap showing the top ten positive marker genes within each cluster. All DEGs are shown in Additional file 7. **D** Heatmap showing average expression of selected T cell function-associated genes of naive markers, cytokines, effector molecules, and Treg markers in each T cell subcluster

To further assess T cell heterogeneity, we applied clustering analysis and identified nine major T cell subsets based on the expressed markers, eliminating clusters 6 and 7, which did not express specific T cell markers (Fig. 5A–C; Additional file 7). We compared the expression levels of selected T cell function-associated genes among the different T cell subtypes and identified four CD8 + T cell subclusters and three CD4 + T cell subclusters (Fig. 5D). Our analysis identified SC-T-1, SC-T-4, SC-T-5_2 as effector CD8 + T cells, and SC-T-3 as naive CD8 + T cells. CD4 + SC-T-2 represented naive CD4 + T cells, while SC-T-5_1 was the effector CD4 + T cells. Treg markers, such as FOXP31 and IL2R, were highly expressed in SC-T-8 cells. We then compared the T cell composition in endometriosis lesions, eutopic endometrium, and normal endometrium, and found, for conventional CD8 + T cells, a significantly higher ratio of naive SC-T-3 in endometriosis lesions (43.48% in endometriosis lesions vs 0.91% in eutopic endometrium and 0.33% in normal endometrium), with decreased effector SC-T-1 (14.68% in endometriosis lesions vs 46.58% in eutopic endometrium and 41.34% in normal endometrium), and effector SC-T-4 (4.43% in endometriosis lesions vs 10.1% in eutopic endometrium and 14.66% in normal endometrium). For conventional CD4 + T cells, the ratio of naive SC-T-2 decreased in endometriosis (20.61% in endometriosis lesions vs. 28.02% in eutopic endometrium and 32.17% in normal endometrium), with increased effector SC-T-5_1 (7.30% in endometriosis lesions and 2.24% in eutopic endometrium vs 1.40% in normal endometrium) (Additional file 1: Fig. S7A, B, Additional file 8).

**Fig. 6:**
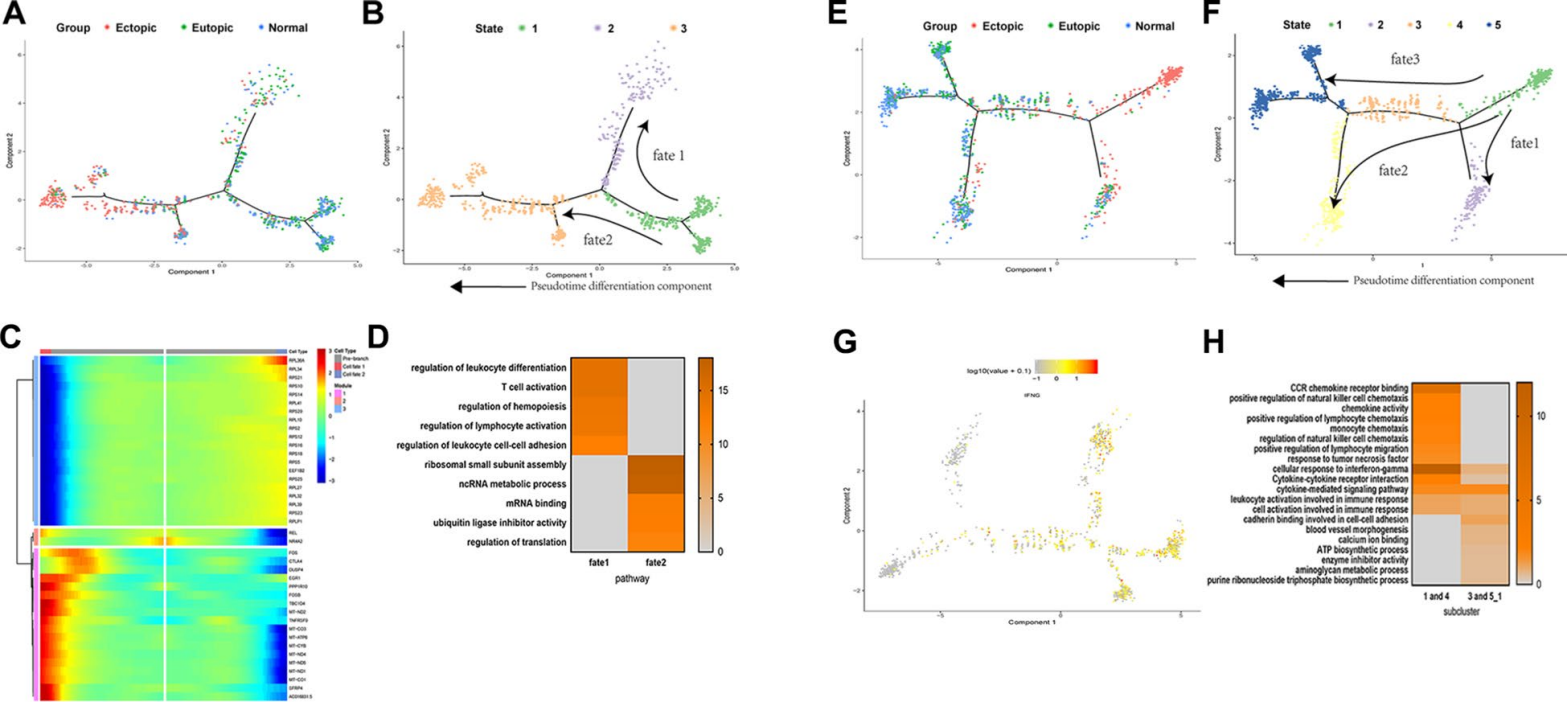
Pseudotime trajectory of T cells clusters in endometriosis lesions, eutopic endometrium and normal endometrium. **A**, **B** Monocle analyses showing the development of CD4 + T cells. **C** Heatmap of different blocks of DEGs along the pseudotime trajectory. The full table of DEGs is shown in Additional file 12. **D** Functional enrichment analyses with GO and KEGG performed with the enriched genes in cells through fate 1 and fate 2. **E**, **F** Monocle analyses showing the development of CD8 + T cells. **G** The expression of IFNG through CD8 + T cells pseudotime trajectory. **H** Functional enrichment analyses with GO and KEGG were compared in characteristic subclusters of CD8 + T cells from endometriosis lesions and endometrium

Monocle trajectory analysis of CD4 + T cells inferred a differentiation trajectory that primarily began with naive SC-T-2 in the endometrium and gradually changed to effector SC-T-5_1 cells with Treg cells branching out. Furthermore, naive CD4 + T cells in the beginning of the trajectory were dominant in the endometrium (Fig. 6A, B). We then compared two development states in the trajectory and found that state 1 was composed primarily of CD4 + T cells from eutopic and normal endometrium; whereas state 2 was occupied by CD4 + T cells derived from endometriosis cysts and endometrium (fate 1). Half of CD4 + T cells in state 3 were from endometriosis lesions (fate 2) (Additional file 1: Fig. S7C). Further, GO and KEGG analyses showed that CD4 + T cells undergoing fate 1 were more related to leukocyte differentiation and activation, while fate 2 CD4 + T cells were associated with homeostatic molecules (Fig. 6C, D), which further confirmed the decreased cell activation of T cells during endometriosis development.

For conventional CD8 + T cells, the differentiation trajectory also exhibited a branched structure, starting with naive SC-T-3 in endometriosis lesions, which then divided into three effector CD8 + T cells (Fig. 6E, F). Comparing these three branches, we found that CD8 + T cells in endometriosis lesions were more likely to go through fate 1, while the percentage of CD8 + T cells in the normal endometrium was higher in fates 2 and 3 (Additional file 1: Fig. S7D). Through changes in the expression of effector genes, such as *IFNG* in the trajectory, we found that CD8 + T cells going through fates 2 and 3 were more effective than CD8 + T cells in fate 1 (Fig. 6G). We also compared GO and KEGG analyses in specific subclusters of CD8 + T cells from endometriosis lesions, eutopic endometrium, and normal endometrium (Fig. 6H) and found decreased cytotoxic T cell populations in endometriosis lesions, suggesting a hindrance in the clearance of the disease lesions [26]. CD4 + FOXP3 + SC-T-8 cells (Treg) are involved in the maintenance of immune homeostasis and secrete the general immunosuppressive cytokine IL-10, which downgraded chronic inflammatory responses and had been shown to inhibit fibrosis in numerous models [27]. The ratio of Treg was decreased in endometriosis lesions, indicating an abnormal immune state. Intriguingly, although the eutopic endometrium contained a high ratio of effector T cells, the percentage of Treg cells that inhibited the proliferation and activation of effector T cells was also higher (7.29% in eutopic endometrium vs. 3.89% in endometriosis lesions and 4.87% in normal endometrium).

#### NK cells

**Fig. 7:**
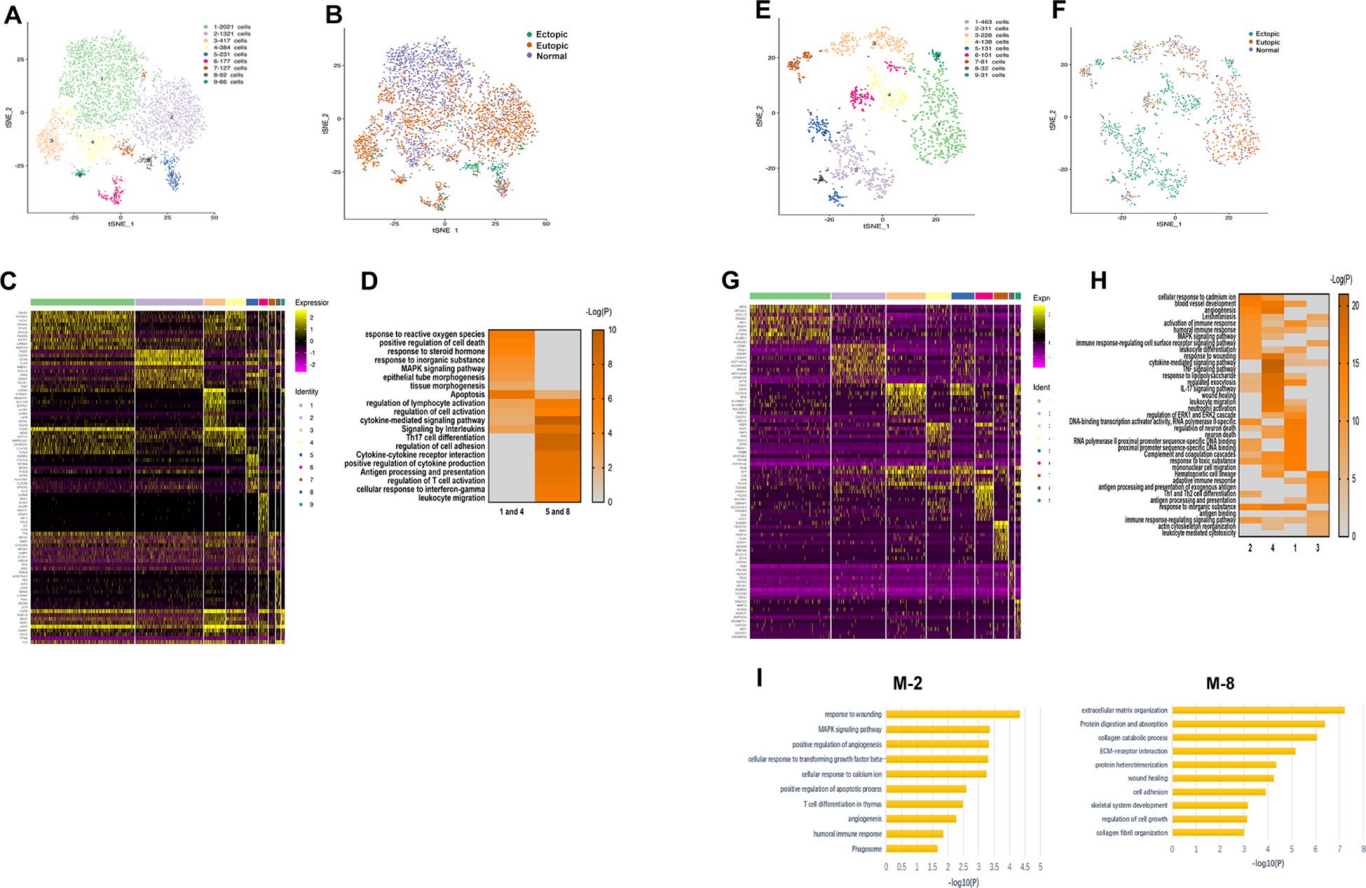
Distinct characteristics of NK and M subpopulations. **A**, **B** Nine subclusters were identified after NK cells were combined and clustered. **C** Heatmap showed the top ten positive marker genes within each cluster. All DEGs are found in Additional file 9. **D** GO and KEGG analysis were compared in characteristic subclusters of NK cells from three groups. **E**, **F** Nine subclusters were identified after M cells were combined and clustered. **G** Heatmap showed the top ten positive marker genes within each cluster. All DEGs are shown in Additional file 10. **H** GO and KEGG analysis were compared in characteristic subclusters of M cells from three groups. **I** Functional enrichment analysis with GO and KEGG analysis of M-2 and M-8

In accordance with our scRNA-seq data, we observed a relatively low proportion of NK cells present in endometriosis lesions (Fig. 4A). Moreover, NK cell-associated gene expression differed among the endometriosis lesions, eutopic endometrium, and normal endometrium (Fig. 4C), consistent with enriched functions (Fig. 4F). To further comprehend the heterogeneity of NK cells, we re-clustered the NK cells and identified nine subclusters with markers shown by heatmap (Fig. 7A–C; Additional file 9). Additionally, we compared the proportion of each subgroup among the samples (Additional file 1: Fig. S7E, F) and found a substantial increase in SC-NK-5 and SC-NK-8, and a decrease in SC-NK-1 and SC-NK-4 in endometriosis lesions compared to the eutopic and normal endometrium.

Via GO and KEGG analyses, we found that up-genes enriched in SC-NK-1 and SC-NK-4 were more associated with cell death regulation and reactive oxygen species, while SC-NK-5 and SC-NK-8 were related to leukocyte migration and activation (Fig. 7D). NK cells in endometriosis lesions were in a more active state but the number of it was too small to exert a sufficient clearance effect.

#### Macrophages/monocytes

We compared gene expression and functions of M cells among three groups and found different gene expressions (Fig. 4D). GO and KEGG analyses of M cells in endometriosis lesions identified pathways enriched in wound healing (Fig. 4G). We then identified nine subgroups of M cells based on their molecular markers (Fig. 7E–G; Additional file 10) and compared the proportion of subclusters among the three groups (Additional file 1: Fig. S7G, H), which showed an increased proportion of CX3CR1 + CCR2 + monocytes/macrophages (SC-M-2) and CXCL5 + IL6 + monocytes/macrophages (SC-M-4) in endometriosis, with a decreased ratio of CXCL12 + monocytes/macrophages (SC-M-1) and CD1 + monocytes/macrophages (SC-M-3). Previous studies have shown that both CX3CL1 and IL6 have a profibrotic effect in vivo [28, 29]. CXCL5 is associated with inflammation, while CXCL12 with homeostasis, which played an important role in modulating immune cell recruitment [12]. We then compared the expressed DEGs of these four specific subclusters using GO and KEGG analyses and found that SC-M-2 and SC-M-4 were related to angiogenesis and wound healing, while SC-M-1 and SC-M-3 were more associated with immune responses (Fig. 7H; Additional file 1: Fig. S8). We also identified specific endometriosis subcluster SC-M-2 that enriched functions including response to wounding and angiogenesis, while SC-M-8 was related to ECM organisation, wound healing, and adhesion (Fig. 7I). These results show that EM-associated M cells are associated with wound healing and tissue remodelling.

We then compared average gene expression of specific genes associated with M1 and M2 macrophages using heatmap and found that the main population of M cells in endometriosis highly expressed CD206 and CD163 (Additional file 1: Fig. S7I), which have been described as M2 genes, indicating impaired immune function and the pro-remodelling nature of endometriosis [30].

### Complex cell–cell communication networks exist in endometriosis lesions, eutopic endometrium, and normal endometrium

**Fig. 8:**
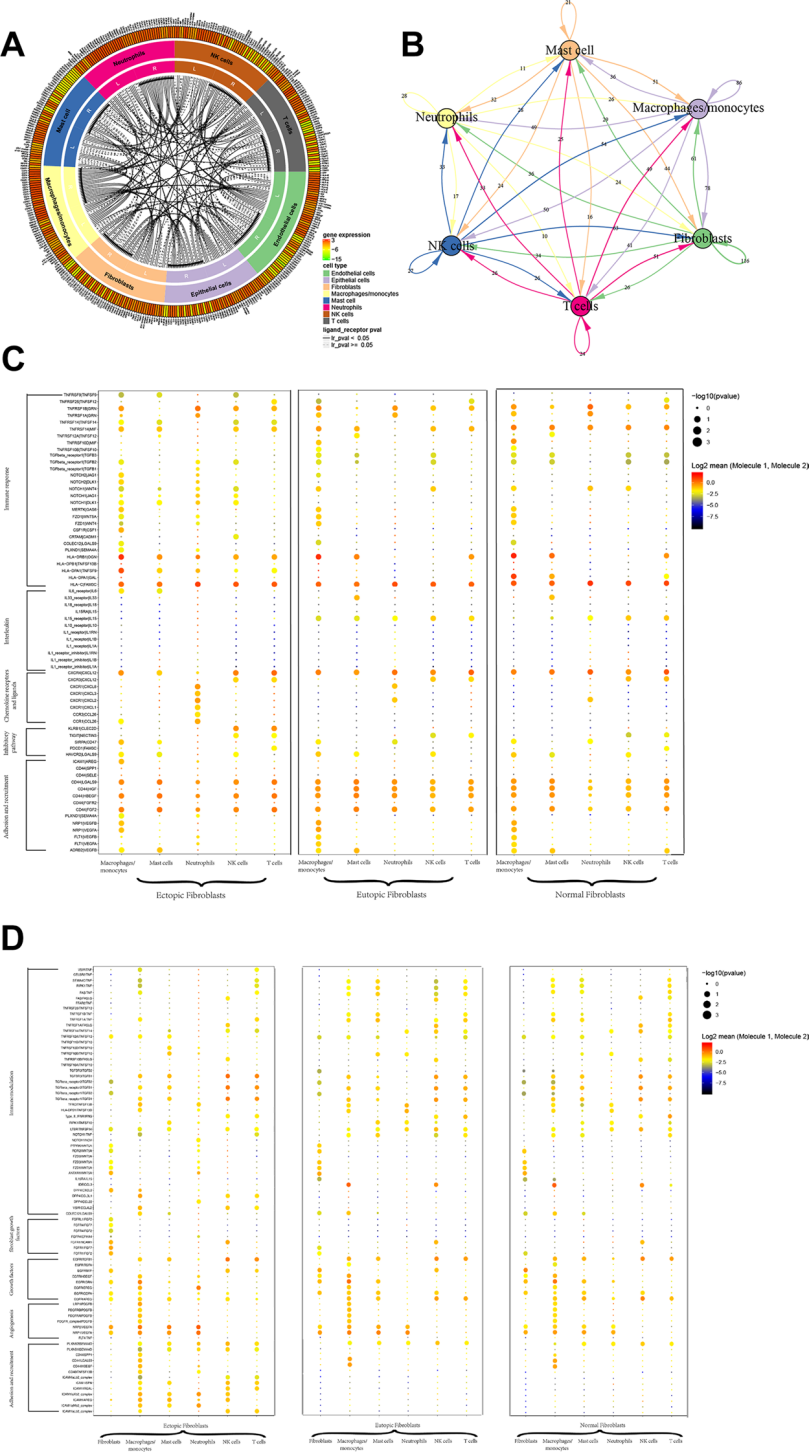
The dense network and multiple cellular connection in endometriosis. **A** Putative signalling between differentially expressed receptors in different cell types and their ligands. Compartments represent cell types, and their preferentially expressed receptors and ligands were labelled along the outer margin. The solid line indicates a significant interaction, while the dashed line, no significant interaction. The communication network of eutopic endometrium and normal endometrium is shown in Additional file 1: Fig. S9. **B** Capacity for intercellular communication among FBs and immune cells. Each line colour indicates the ligands expressed by the cell population represented in the same colour (labelled). The lines connected to the cell types that expressed the cognate receptors. The line thickness was proportional to the number of ligands when cognate receptors were present in the recipient cell type. **C**, **D** Overview of selected ligand–receptor interactions of FBs, macrophages, NK cells, T cells, mast cells and neutrophils. P values are indicated by circle size, with the scale to the right (permutation test)

To systematically assess the associated complex cellular responses, we attempted to map ligand–receptor interactions with our scRNA-seq data to better understand cellular behaviour and response to neighbouring cells in endometriosis. We considered the expression levels of ligands and receptors within each cell type and predicted molecular interactions between cell populations via specific protein complexes. We then generated a potential intercellular communication network among all cells in the three groups separately (Fig. 8A; Additional file 1: Fig. S9A). Broadcast ligands for which cognate receptors were detected and manifested broad communication between immune cells and FBs (Fig. 8B; Additional file 1: Fig. S9B).

Interactions of chemokines between neutrophils and fibroblasts were commonly observed. FBs in endometriosis cells produced higher levels of inflammatory factors IL-6 in ectopic endometrium than in eutopic endometrium from endometriosis women, while the corresponding receptors were expressed in macrophages/monocytes and mast cells. We also identified putative inhibitory interactions between endometriosis immune cells and ectopic FBs rather than eutopic FBs, including KLRB1, TIGIT, and PDCD1, which were highly expressed by NK and T cells, and potentially bind to CLEC2D, NECTIN3, and FAM3C, which were expressed by FBs (Fig. 8C).

Notably, the immune cells in ectopic lesions expressed relatively higher levels of adhesion molecules, especially in M cells, as well as higher levels of growth and angiogenesis factors while the corresponding receptors are widely expressed in FBs, which could enhance the adhesion and growth of FBs (Fig. 8D).

## Discussion

The origin of endometrial-like tissues that constitute endometriosis remains controversial, however, it is generally accepted that endometriosis is a normal phenomenon, while many of the small lesions tend to be spontaneously inactive [9]. Although accumulating evidence suggests cell heterogeneity between endometriosis lesions and endometrium [31–33], our understanding remains limited by the incomplete knowledge regarding cell type complexity. In this study, we investigated the heterogeneity of cells and described the composition of cellular subtypes, then constructed a comprehensive single-cell transcriptome atlas of the multicellular ecosystem of endometriosis based on our new data. We also provided insights into the disease interaction network.

Our scRNA-seq based analysis revealed detailed changes in cell subtypes and localised expression of pathogenic genes to specific cell subpopulations. Although the characteristics of the eutopic endometrium and normal endometrium were generally similar, differences were apparent in the cell subtypes, reflected by cell composition and gene expression. This supports the hypothesis that the important defect might be partially rooted in the eutopic endometrium of women with endometriosis. Abnormal eutopic endometrium is important for the onset of ectopic diseases. This complex cell composition also explained why previous whole-transcriptome sequencing of eutopic endometrium versus normal endometrium, based on whole samples with various components, generated conflicting results [33].

Analysis of biological processes implicated in endometriosis through scRNA-seq data demonstrated the unique role of this approach in understanding disease pathogenesis and could explain the complexity associated with endometriosis signalling. The pathobiology of endometriosis involves the aberrant transformation of the disease developmental pathways, including TGF-β, MAPK, Rho-Rack, NF-κB, and JAK-STAT pathways, as well as those associated with EMT [34]. Our single-cell RNA-seq data were consistent with the emerging concept that these pathways are associated with the development of the disease, allowing us to assign the expression of genes associated with these pathways to specific cellular populations within the endometriosis lesion [35]. Specifically, we noticed that the main cell population, FBs, played a decisive role in the disease process via genetic alteration. We used scRNA-seq to distinguish FBs and determined the disease associated FB subtypes. FBs in endometriosis lesions exhibit obvious genetic differences compared to eutopic and normal endometrium, which may explain the occurrence and development of endometriosis. Previous studies have also emphasised the significance of fibrosis in endometriosis [30]. Our scRNA-seq data revealed an EM-associated FBs development pathway and found a similar trajectory of FBs between ectopic and eutopic endometrium. Moreover, retrograde menstruation has been reported as a key factor in endometriosis pathogenesis [36, 37]. Meanwhile, by reconstructing a clonal evolution tree based on somatic mutations, Suda et al. suggested that epithelial cells of ovarian endometriosis are descendants of uterine endometrial epithelium [38]. Our data also demonstrated overlapping FB trajectories and similar gradual changes in the gene pathogenic spectrum between ectopic and eutopic endometrium, supporting the hypothesis that the stroma of endometriosis might originate from eutopic endometrium [7, 9, 39, 40] and pathogenic changes of FBs played a key role in this process. Hence, endometriosis appeared to not be simply transplanted endometrium, but rather a gradual process of transformation of many important pathogenic genes with subsequent implantation and transition from endometrium to endometriosis [1]. Our analysis provides a reference for subsequent analyses of endometriosis origin. Nevertheless, we could identify the regulatory mechanism underlying the altered processes in FB trajectory, which might be related to epigenetic modification [41].

As a hormone-dependent disease, an aberrant immune-endocrine microenvironment was determined to be ideal for the growth and survival of ectopic lesions [12]. We found higher expression of oestrogen receptor b (ERb) in endometriosis lesion FBs which could promote ectopic lesion growth through enhanced proliferative activity and reduced apoptotic signalling [42]. Meanwhile, progesterone receptor (PGR), which downregulates oestrogen receptors, was decreased in endometriosis FBs [1]. We also found that aromatase (CYP19A1) and steroidogenic acute regulatory protein (StAR), were highly expressed in endometriosis FBs, which could increase oestrogen levels and expression of StAR both of which contribute to the development of the disease. Taken together, the results indicated that the hormonal environment of endometriosis lesions was dysregulated (Additional file 1: Fig. S10A).

A meta-analysis reported that endometrial environment in women with stages I-II could be more pro-inflammatory than in women with stages III–IV [43]. However, our study only included samples from women with stages III-IV. We found decreased cytotoxicity and cell activation of T cells in endometriosis lesions, suggesting hindrance in the clearance of the disease lesions [26]. Meanwhile, eutopic endometrium had higher Treg proportion, suggesting that the effectiveness of T cells in eutopic endometrium was inhibited. We also observed different characteristics of NK cell subclusters among the three groups with fewer NK cells in endometriosis lesions, which could aggravate the immune microenvironment. A previous study proposed the NK cell theory of endometriosis, presuming that endometriotic cells are natural NK target cells [44]. Our results also suggested an impaired ability for NK cells to clear cells from endometriosis lesions, leading to lesion growth. Moreover, endometriosis has also been regarded as a disease of macrophages [9, 45], which are key effectors in phagocytosing pathogens and acted as antigen-presenting cells, as well as participate in tissue regeneration, angiogenesis, and wound healing [46]. Research showed that endometria obtained from women with stages III-IV endometriosis contained predominantly M2 macrophages and NKT [43]. Increased M2 in endometriosis lesions would downregulate immune response and induce angiogenesis, as well as tissue repair that favoured the development and growth of endometriotic lesions [47, 48]. In addition, M2 could inhibit the function of NK cells [49], thereby limiting their effector function and further promoting immune imbalance in endometriosis lesions. M2 cells were also involved in nerve growth, suggesting a role in endometriosis-related pain [50]. Taken together, dysfunctional immune responses played a substantial role in the pro-inflammatory phenotype characteristic of endometriosis lesions. Symons et al. concluded that innate and adaptive immune cells from patients with endometriosis contributed to disease pathophysiology [12, 20]. Similarly, a recent report used scRNA-seq to reveal a distinct immune environment in peritoneal fluid derived from women with endometriosis and identify specific subpopulations of T cells [4]. Specifically, the peritoneal fluid of patients with endometriosis was an inflammatory microenvironment containing an increased concentration of macrophages and activated macrophages, as well as decreased NK cell activity [51]. The generation of peritoneal fluid in patients with endometriosis might be caused by the disease status. For instance, endometriosis lesions might cause local stimulation in the abdominal cavity, thereby increasing vascular permeability and promoting the production of peritoneal fluid. Substances, such as cytokines, growth factors, and angiogenetic factors in peritoneal fluid would, in turn, stimulate the occurrence and development of endometriosis lesions [51–53]. However, to date, there have been no studies on the immune environment using scRNA-seq in ovarian endometriosis lesions. In this study, we identified the most relevant immune components of disease pathogenesis, as well as interactions among them using scRNA-seq.

This study expands our understanding of the immune microenvironment presented within the ovarian lesions in patients with endometriosis and provides several important findings. First, we identified a microenvironment with impaired immune function. Retrograde menstruation induces innate immune activation, including increased numbers of macrophages, which is likely the primary step in the pathophysiology of endometriosis [54]. Our results quantitatively confirmed that macrophages were the major cell population with increased pro-fibrosis and tissue repair ability in endometriosis lesions. In addition, we found that M2 macrophages in endometriosis displayed pro-inflammatory signatures that expressed CD40 and CD64, indicating a hybrid of M1 and M2 cell profiles within endometriosis lesions. This suggests that macrophages could adopt a phenotypic ‘switch’ between M1 and M2 activation [48, 55]. Furthermore, we observed a decreased number of NK cells and depleted T cells activity, which was associated with the development of more frequent and larger lesions of endometriosis cysts [56]. In summary, endometriosis was an immune-related disease that initiated the inflammatory system and led to the formation of peritoneal fluid when the disease progressed.

We hypothesized that the immune dysfunction might favour the survival and implantation of ectopic endometrium, while ECM remodelling and angiogenesis might induce metaplasia of the peritoneum or development of Müllerian remnants [9, 10, 57]. Moreover, this altered inflammatory microenvironment could favour the implantation and development of endometriosis lesions from refluxed endometrium [7]. Therefore, investigating the properties of immune cell subtypes and their dynamics may provide insights into previously unexplained phenomena. Taken together, these data improved our understanding of immune cell heterogeneity in the immunopathogenesis of endometriosis and supported the immune-related hypothesis.

Communication among cells in endometriosis lesions played a key role in the development of this disease. In this study, we observed a high expression of inflammatory associated ligands in endometriosis FBs, suggesting an imbalanced immune environment in the disease lesions. Besides, inhibitory interactions between ectopic FBs and immune cells suggested limited activation of immune cells in endometriosis lesion. High expressions of FB receptors associated with pro-fibrosis and adhesion, such as FGFR1, FGFR4, ICAM, and CD44, were activated by ligands in immune cells, suggesting that alternate endometriosis immune cells played an important role in promoting lesion development.

This study has some limitations. First, as one of the most complex tissues in women, the uterus consisted of many cell types. Due to the limited sample size, we only included a portion of the functional endometrium layers in our analysis, which may not fully reflect the characteristics of the endometrium. The small number of samples might have affected the final conclusion. However, the repeatability of the three samples in each group was relatively high according to our results (Additional file 1: Fig. S10B, C) and we were able to identify many of the meaningful features and changes that could provide further insights into the disease even with limited samples. Second, due to the limited sample size, we could not statistically analyse samples stratified by symptoms, menstruation phase, and disease stage. Endometriosis has a heterogeneous clinical presentation affected by several factors, and hence further larger datasets from normal and diseased samples should be analysed to clarify the stratified changes of these factors, to allow the current atlas of the cellular types to be expanded. Third, as ovarian endometrium was adjacent to the ovary, it was inevitably affected by ovarian tissue, including ovarian paracrine pathways, so it would affect us to know primary change in the phenotype or genotype occurred in endometriosis lesion, which could not be avoided at present. Fourth, computational approaches for analysing scRNA-seq datasets, unbiased identification of cell types, and integrative analysis were in their seedtime. As such, they were biased by the batch effect or ambient RNA due to the true biological effect associated with inter-subject variability or disease conditions. The analyses and annotations of cell types presented here were not exhaustive, and could, therefore, not fully differentiate between similar cells. We expected that this would be improved in the future, particularly with the help of computational tools developed by research consortia, such as the Human Cell Atlas [58]. With regard to the gene changes found in this study, RNA in situ hybridisation and immunohistochemistry could verify gene changes. In addition, future functional validations, such as direct cytokine measurements or cytotoxicity assays, could provide more data on endometriosis.

In summary, our study provided a scalable insight into the origin of endometriosis as well as related pathogenic genes, which could be considered as future targets for treatment by blocking their actions.

## Conclusion

Considering the cellular complexity and dynamics of the disease, advanced understanding of the functional contribution to disease for each cell subtype would help to explain the pathogenic mechanisms involved in endometriosis. To this end, our study unbiasedly scrutinised ovary endometriosis at single-cell resolution. Our findings demonstrated the cellular complexity of endometriosis development and suggested meaningful trajectory that represented disease progression. Each cell type possessed multiple cell subtypes with distinct molecular features, and displayed different composition in their subtypes among endometriosis lesion, eutopic endometrium and normal endometrium. Besides, cell–cell interaction analyses highlighted the imbalanced immune environment in endometriosis; the immune cells in endometriosis influence the development of the disease. Our study provided an insight into the origin of endometriosis as well as related pathogenic genes which could potentially be treatment targets against endometriosis.

## Methods

### Patient samples

Three patients diagnosed with endometriosis by laparoscopy and pathological examination were enrolled in this study. Normal endometrium tissues from three healthy patients were also collected as controls. All patients presented regular menstrual cycles and had not received any hormone therapy within the last three months. The median age of patients with endometriosis was 31.6. Moreover, any patients presenting with adenomyosis, or other malignant tumours were excluded. Their clinical characteristics were summarised in Table 1. Fresh samples were obtained at the time of cystectomy or shave and were immediately placed into tissue protection fluid. All clinical samples including three ectopic endometrium from endometriosis lesion, three eutopic endometrium from women with endometriosis, and three normal endometria from women without endometriosis were collected at the Department of Obstetrics and Gynecology, Women’s Hospital, School of Medicine, Zhejiang University.

### Preparation of single-cell suspensions

Samples for scRNA-seq were placed on ice, washed with phosphate-buffered saline, cut into pieces < 1 mm^3^, and then put into 3 mL Dulbecco’s modified Eagle’s medium containing collagenase IV (1 μg/mL; Thermo Fisher Scientific). The samples were incubated at 37 °C for 60 min in automatic oscillator and then filtered twice using 40-μm nylon mesh. Following centrifugation (1000 × rpm, 4 °C, and 5 min), the supernatant was discarded, and the cell pellet was then resuspended in 1 mL fetal bovine serum. After lysing the red blood cells and removing dead cells, the cells were counted and assessed for viability using Trypan blue staining with a hemocytometer [59]. During the dissociation procedure, the cells were kept on ice whenever possible to avoid dissociation-associated artefacts. A positive signal for a dissociation signature that reflects dissociation-associated changes in gene expression was obtained in < 1% of the cells.

### Droplet-based scRNA-seq

A Chromium Single-cell 3-Library was constructed using the Chromium Single-cell 3-Library, Gel Bead & Multiplex kit, and Chip kit according to the manufacturer’s instructions. Cell suspensions were loaded onto a chromium single-cell chip along with reverse transcription master mix and single-cell 3-gel beads, with ~ 5000–10,000 single cells per reaction. The samples were processed using 10 × Genomics V3 barcoding chemistry kits. Following cell lysis, first-strand cDNA synthesis and amplification were carried out according to the manufacturer’s instructions. Libraries were sequenced using an Illumina novaseq6000.

### Single-cell gene expression quantification

The Cell Ranger software pipeline (version 3.1.0) provided by 10 × Genomics was used to demultiplex cellular barcodes, map reads to the genome and transcriptome using the STAR aligner, and down-sample reads as required to generate normalised aggregate data across samples, producing a matrix of gene counts versus cells [59, 60]. We processed the UMI count matrix using the R package Seurat (version 3.0.0) to remove low-quality cells and likely multiples. We then applied a criterion to filter out cells with UMI/gene numbers outside the limit of mean value ± twofold of standard deviation, assuming a Gaussian distribution of each cell’s UMI/gene numbers. Following visual inspection of the distribution of cells by the fraction of mitochondrial genes expressed, we further discarded low-quality cells where > 25% of counts belonged to mitochondrial genes. Library size normalisation was performed in Seurat on the filtered matrix to obtain the normalised count.

### Identification of the major cell types and their subtypes

The top variable genes across single cells were identified. Briefly, the average expression and dispersion were calculated for each gene, and genes were subsequently placed into several bins based on expression. To remove the batch effects in single-cell RNA-sequencing data, the mutual nearest neighbours (MNN) presented by Haghverdi et al. was performed with the R package bachelor [61]. Cells were clustered based on a graph-based clustering approach and were visualised in 2-dimensions using t-SNE. The likelihood ratio test that simultaneously tested for changes in mean expression and in the percentage of expressed cells was used to identify significantly DEGs between clusters. Here, we used the R package SingleR, a novel computational method for unbiased cell type recognition of scRNA-seq to infer the cell of origin of each single-cell independently and to identify cell types.

We then annotated the clusters based on the average expression of curated gene sets of the following major cell types: epithelial cells (*EPCAM, KRT19, KRT18, KRT5, KRT15*), macrophages/monocytes (*CD68, MS4A4A, MS4A7*), T cells (*CD2, CD3D/E/G*), MAST cells (*CD117, TPSB2, TPSAB1*), NK cells (*TRDC, KLRC1*), neutrophils (*CD16, CD66*), ECs (*AQP1, MYCT1, CDH5, PECAM1*), and FBs (*COL1A1, COL3A1, COL1A2*)(representative marker reference from: http://biocc.hrbmu.edu.cn/CellMarker/ and https://panglaodb.se/search.html) [60].

Next, we performed cluster-based doublet exclusion. After doublet removal, we performed steps (normalisation, dimensionality reduction, and clustering) to identify the major cell types among the remaining cells. To identify subclusters within each major cell type, the cells belonging to each cell type were reanalysed separately; we performed dimensionality reduction, and clustering as described above for each of the major cell types. Then, the subclusters were annotated to cell subtype by the average expression of the corresponding curated gene sets.

DEGs were identified using the Seurat package. A P value < 0.05 and |log2foldchange|> 0.58 was set as the threshold for significantly differential expression. GO enrichment and KEGG pathway enrichment analyses of up-expressed genes were performed using Metascape (http://metascape.org/) and DAVID Bioinformatics Resources 6.8 (https://david.ncifcrf.gov/).

### Trajectory analysis

Trajectory analysis was performed separately for the fibroblasts, CD8 + T cells, and CD4 + T cells using Monocle 2 (version 2.8.0) [62]. We then conducted differential gene expression analysis of the studied cells using the differential Gene Test function to identify significant genes (BH-corrected P < 0.01). Cell ordering was performed on these genes in an unsupervised manner. Trajectory construction was then performed after dimensionality reduction and cell ordering with default parameters.

### Cell–cell communication analysis

To investigate potential interactions across different cell types in the endometriosis lesions and endometrium, cell–cell communication analysis was performed using CellPhoneDB, a publicly available repository of curated receptors and ligands and their interactions [62]. CellPhoneDB analysis was performed using CellPhoneDB Python (package 2.0.0). Single-cell transcriptomic data of cells annotated as epithelial cells, macrophages/monocytes, T cells, MAST cells, NK cells, neutrophils, ECs, and fibroblasts were input into CellPhoneDB for cell–cell interaction analysis. Enriched receptor-ligand interactions between two cell types were derived based on the expression of a receptor by one cell type and the expression of the corresponding ligand by another cell type. We then identified the most relevant cell type-specific interactions between ligands and receptors. Pairwise comparisons were performed between the included cell types. We first randomly permuted the cluster labels of all cells 1,000 times to determine the mean of the average receptor and ligand expression levels of the interacting clusters. This generated a null distribution for each receptor-ligand pair. By calculating the proportion of the means that were higher than the actual mean, a P value for the likelihood of cell type specificity of the corresponding receptor-ligand complex was obtained. We then selected interactions that were biologically relevant.

### Immunofluorescence analysis

Four-micrometre tissue sections from formalin-fixed, paraffin-wax-embedded human endometriosis tissues and endometrial tissues were dewaxed, rehydrated, and subjected to high-temperature antigen retrieval. The tissues were incubated with blocking antibody diluent at room temperature for 2 h, and then incubated overnight at 4 °C with the following primary antibodies: anti-vimentin (mouse, ab8978, dilution 1:1000; Abcam), anti-CXCL8 (rabbit, sc-8427, dilution 1:150; Santa Cruz), anti-C7 (rabbit, ab126786, dilution 1:150; Abcam), anti-S100A10 (rabbit, sc-81153, dilution 1:150; Santa Cruz), anti-C3 (rabbit, ab200999, dilution 1:150; Abcam), and anti-StAR (rabbit, sc-166821, dilution 1:150; Santa Cruz). The slides were then incubated with secondary antibody (HRP polymer, anti-mouse/rabbit IgG, Abcam) at room temperature for 1 h. Nuclei were stained with 4-6-diamidino-2-phenylindole (DAPI, ab104139, Abcam) after all antigens had been labelled and then imaged.

### Isolation and culture of FBs

Endometriosis endometrial tissues were isolated as previously described [63] and cultured with DMEM/F12 medium (Biological Industries, USA) that contained 10% fetal bovine serum (Invitrogen, USA) as well as 1% penicillin–streptomycin (Biological Industries, USA) at 37 °C in 5% CO_2_ and 95% air atmosphere.

### Small-interfering RNA transfection and Western blot analysis

FBs were transfected with *StAR* small-interfering RNA (siRNA; si-*StAR*) and negative control siRNA (si-Ctrl), which were produced by Genepharma Corporation (Shanghai, China) by Lipofectamine RNAiMAX (Invitrogen, USA) in six-well plates. After transfection for 72 h, total protein was isolated, and western blot analysis was used to assess transfection efficiency according to the manufacturer’s instructions.

The siRNA strands were shown as follows:

si-Ctrl: 5′-ACGUGACACGUUCGGAGAATT-3′ (antisense),

5′-UUCUCCGAACGUGUCACGUTT-3′ (sense);

StAR-Homo-296: 5′-GGAGCUCCUACAGACACAUTT-3′ (antisense),

5′-AUGUGUCUGUAGGAGCUCCTT-3′ (sense);

Antibodies used in our research were rabbit antibody against StAR (dilution 1:10,000, ab133657, abcam), mouse antibody against Tubulin (dilution 1:5000, AT819, Beyotime), and anti-mouse/anti-rabbit secondary antibodies (dilution 1:10,000, WD-0990/WD-GAR007, DawenBiotech).

### Functional experiment

Transwell migration and matrigel invasion assays, wound scratch assay, CCK8 assay and flow cytometric analysis were conducted in accordance with previous work [63].

### Statistics

All statistical analyses were performed using GraphPad Prism 8 and R software (http://www.rproject.org). We used the R Base package with default parameters to generate box plots. The Beanplot R package was used to generate violin plots, with the data distribution bandwidth evaluated by kernel density estimation. We did not display each data point in all box and violin plots because the large number of data points would obscure the overall distribution. The results from at least three independent experiments were presented as mean ± SEM. A two-sided paired or unpaired Student’s *t*-test and unpaired Wilcoxon rank-sum test were used where indicated. P < 0.05 was considered to indicate statistical significance.

## Supplementary Information

**Additional file 1: Figure S1.** Study flow chart. Single-cell RNA-sequencing. **Figure S2 a-g.** Enriched functions of FBs, Eps, ECs, T cells, NK cells, M cells, ast cells and neutrophils by GO and KEGG analyses. **Figure S3. Single-cell RNA-seq analysis revealed distinct characterization of cell populations in endometriosis lesions, eutopic endometrium and normal endometrium.** (A) Similarity analyses of each cell population in endometriosis lesions, eutopic endometrium and normal endometrium. (B) Differential expression analysis was performed comparing whole cells from endometriosis lesions, eutopic endometrium and normal endometrium. (C) Representative significantly enriched GO and KEGG processes were performed with the significantly upregulated genes in endometriosis lesions, eutopic endometrium and normal endometrium. (D) Expression of selected pathway genes were shown for each cell of endometriosis, eutopic endometrium, or normal endometrium origin. Dot size corresponded to the percentage of cells in the cluster expressing a gene, and dot color corresponded to the average expression level for the gene in the cluster. **Figure S4.** DEG analysis compared cells from endometriosis lesions, eutopic endometrium, and normal endometrium within ECs and Eps by heatmap. **Figure S5.** Immunofluorescence in cells showed the *C3, C7, StAR* and *S100A10* positive FBs in endometriosis lesions. **Figure S6.** (A) Contribution of each group to each cell state on Figure 2E. The majority of state 1 was occupied by FBs from the eutopic endometrium and normal endometrium. State 2 was primarily contained by ectopic and eutopic endometrium FBs, while state 3 contained FBs from all three groups. (B) Relative contributions of FBs from13 subclusters to each group. (C) Relative contributions of FBs from three groups to each cluster, as shown by the t-SNE plot. (D)Western blot analysis reflected si-*StAR* transfection efficiency. (E) Scratch wound healing determined the ability of migration between si-Ctrl and si- *StAR* FBs. (F) Transwell assays were used to compare the effects of si- *StAR* on migration and invasion. (G) Proliferative ability between si-Ctrl and si- *StAR* FBs. (H) Representative images of flow cytometric cell cycle analysis. (I) Apoptosis of si-Ctrl and si- *StAR* FBs by flow cytometric. **Figure S7.** (A) Bar plot demonstrated the relative ratio of each subcluster to the entire T cell population. (B) Relative contributions of T cells from three groups to each cluster. (C) Contribution of each CD4+ T cells group to each cell state on Figure 6B. The majority state 1 was occupied by CD4+ T cells from eutopic endometrium and normal endometrium. State 2 contained CD4+ T cells from all three groups while half of CD4+ T cells in state 3 were from endometriosis lesions. (D) Contribution of each CD8+ T cells group to each cell state on Figure 6F. The majority of state 1 was occupied by CD8+ T cells from endometriosis lesions. Eutopic endometrium and normal endometrium and half of CD8+ T cells in state 2 were from endometriosis lesions. State 3 and state 4 contained T cells from all three groups while normal endometrium CD8+ T cells accounted for most of state 4. State 5 was mostly occupied by CD8+ T cells from eutopic and normal endometrium. (E) Bar plot demonstrated the relative ratio of each subcluster to three NK cell groups. (F) Relative contributions of T cells from three groups to each cluster. (G) Bar plot demonstrated the relative ratio of each subcluster to three M cell groups. (H) Relative contributions of M cells from three groups to each cluster. (I) Heatmap showed average expression of specific genes of M1 and M2 including CD64, CD40, CD86, CD163 and CD206. **Figure S8.** Functional enrichment analysis with GO and KEGG analyses of M-1, M-3, M-4, M-5, M-6, M-7, and M-9. **Figure S9. **The dense network and multiple cellular connection in eutopic endometrium and normal endometrium. (A) Putative signaling between differentially expressed receptors in different cell types and their ligands. Compartments represented cell types, and their preferentially expressed receptors and ligands were labeled along the outer margin. The solid line indicated that there was a significant interaction, and the dashed line indicated that it was not significant. (B) Capacity for intercellular communication between FBs and immune cells. Each line color indicated the ligands expressed by the cell population represented in the same color (labeled). The lines connected to the cell types that expressed the cognate receptors. **Figure S10.** (A)Heatmap of average gene expression of ESR2, PGR, StAR, CYP19A1. (B) Relative contributions of each cluster to each sample. (C) t-SNE plots of cells from each sample profiled in this study, with each cell color coded to indicate the associated cell types.

**Additional file 2.** Characteristics of samples included in this study.

**Additional file 3.** Differentially expressed genes of FBs, M cells, NK cells, T cells, ECs, Eps, mast cells, and neutrophils.

**Additional file 4.** Differentially expressed genes of all FBs subclusters.

**Additional file 5.** Differentially expressed genes of FBs along the pseudotime trajectory.

**Additional file 6.** The relative ratio of immune cells in endometriosis lesions, eutopic endometrium, and normal endometrium.

**Additional file 7.** Differentially expressed genes of all T cells subclusters.

**Additional file 8.** The relative ratio of T cells subclusters in endometriosis lesions, eutopic endometrium, and normal endometrium.

**Additional file 9.** Differentially expressed genes of all NK cells subclusters.

**Additional file 10.** Differentially expressed genes of all M cells subclusters.

**Additional file 11.** Differentially expressed genes of FB clusters among endometriosis lesions, eutopic endometrium, and normal endometrium.

**Additional file 12.** Differentially expressed genes of CD4+ T cells along the pseudotime trajectory.

## Data Availability

All relevant data supporting the findings of this study are available in the article, Additional files, or from the corresponding authors on reasonable request.

## Abbreviations

scRNA-seq: Single-cell RNA-sequencing
siRNA: Small-interfering RNA
si-Ctrl: Control siRNA
UMI: Unique molecular identifier
t-SNE: T-distributed stochastic neighbour embedding
FBs: Fibroblasts
ECs: Endothelial cells
Eps: Epithelial cells
NK: Natural killer cells
M: Macrophages/monocytes
DEGs: Differentially expressed genes
GO: Gene ontology
KEGG: Kyoto Encyclopedia of Genes and Genomes
EMT: Epithelial-to-mesenchymal transition
CCK8 assay: Cell counting kit-8 assay
