# Supplementary figures and images for: Single-cell transcriptomic analysis of endometriosis provides insights into fibroblast fates and immune cell heterogeneity
Source: Cell Biosci. 2021 Jul 7;11:125. doi: 10.1186/s13578-021-00637-x (PMC8261960; doi:10.1186/s13578-021-00637-x)

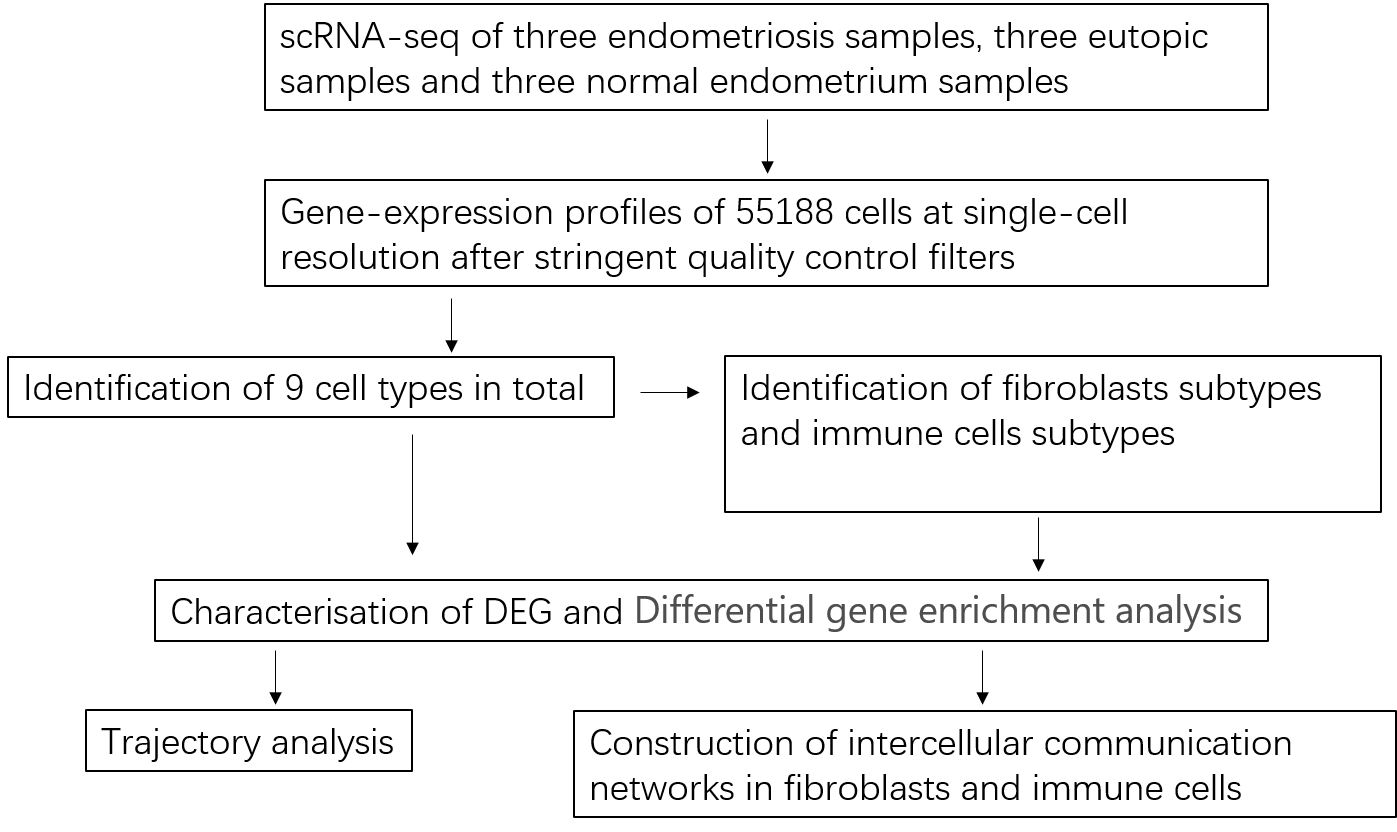

Supplement: Supplementary file 1 — Additional file 1: Figure S1. Study flow chart. Single-cell RNA-sequencing. Figure S2 a-g. Enriched functions of FBs, Eps, ECs, T cells, NK cells, M cells, ast cells and neutrophils by GO and KEGG analyses. Figure S3. Single-cell RNA-seq analysis revealed distinct characterization of cell populations in endometriosis lesions, eutopic endometrium and normal endometrium. (A) Similarity analyses of each cell population in endometriosis lesions, eutopic endometrium and normal endometrium. (B) Differential expression analysis was performed comparing whole cells from endometriosis lesions, eutopic endometrium and normal endometrium. (C) Representative significantly enriched GO and KEGG processes were performed with the significantly upregulated genes in endometriosis lesions, eutopic endometrium and normal endometrium. (D) Expression of selected pathway genes were shown for each cell of endometriosis, eutopic endometrium, or normal endometrium origin. Dot size corresponded to the percentage of cells in the cluster expressing a gene, and dot color corresponded to the average expression level for the gene in the cluster. Figure S4. DEG analysis compared cells from endometriosis lesions, eutopic endometrium, and normal endometrium within ECs and Eps by heatmap. Figure S5. Immunofluorescence in cells showed the C3, C7, StAR and S100A10 positive FBs in endometriosis lesions. Figure S6. (A) Contribution of each group to each cell state on Figure 2E. The majority of state 1 was occupied by FBs from the eutopic endometrium and normal endometrium. State 2 was primarily contained by ectopic and eutopic endometrium FBs, while state 3 contained FBs from all three groups. (B) Relative contributions of FBs from13 subclusters to each group. (C) Relative contributions of FBs from three groups to each cluster, as shown by the t-SNE plot. (D)Western blot analysis reflected si-StAR transfection efficiency. (E) Scratch wound healing determined the ability of migration between si-Ctrl and si- [file 13578_2021_637_MOESM1_ESM.zip › 13578_2021_637_MOESM1_ESM.jpg]

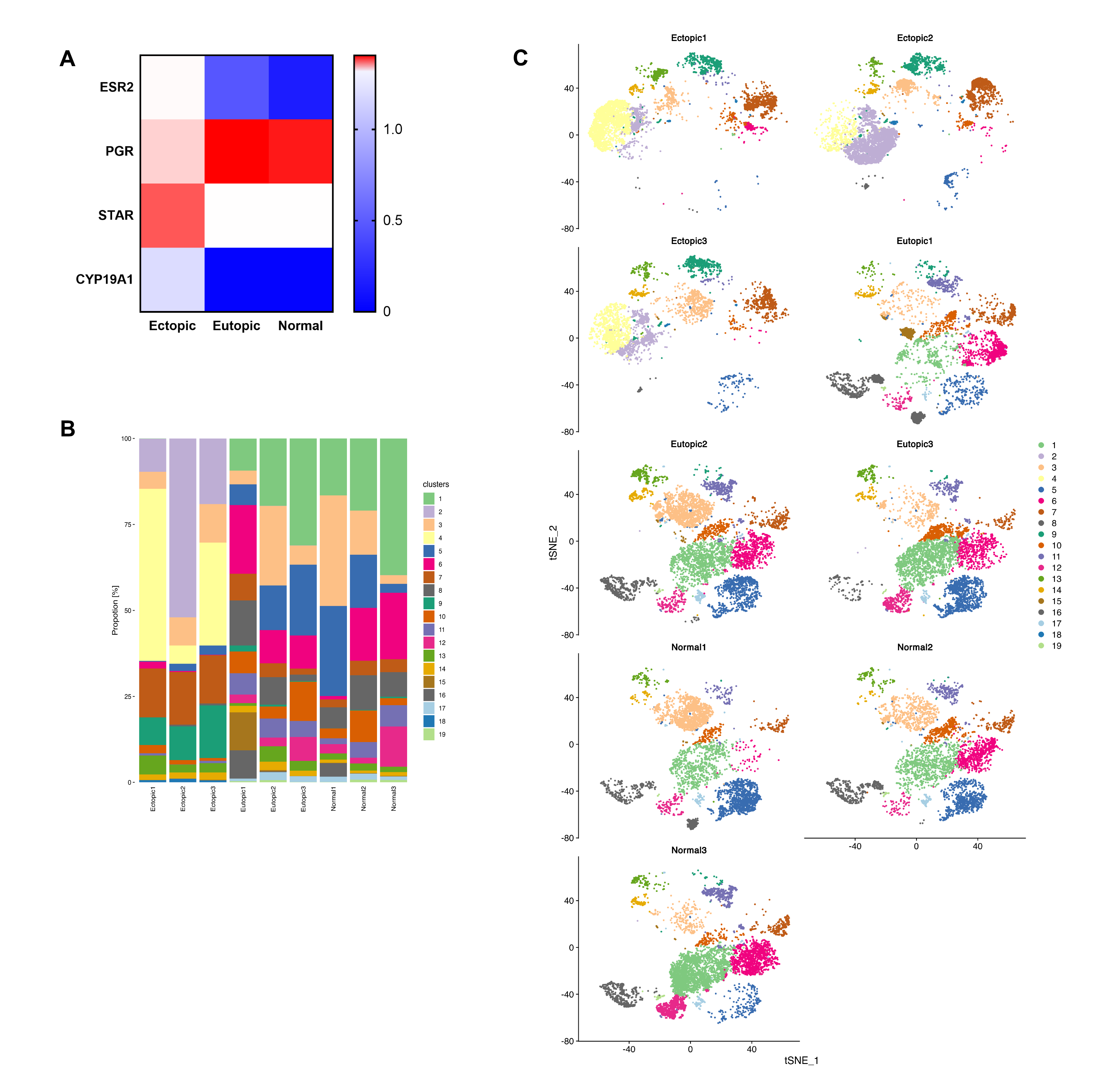

Supplement: Supplementary file 1 — Additional file 1: Figure S1. Study flow chart. Single-cell RNA-sequencing. Figure S2 a-g. Enriched functions of FBs, Eps, ECs, T cells, NK cells, M cells, ast cells and neutrophils by GO and KEGG analyses. Figure S3. Single-cell RNA-seq analysis revealed distinct characterization of cell populations in endometriosis lesions, eutopic endometrium and normal endometrium. (A) Similarity analyses of each cell population in endometriosis lesions, eutopic endometrium and normal endometrium. (B) Differential expression analysis was performed comparing whole cells from endometriosis lesions, eutopic endometrium and normal endometrium. (C) Representative significantly enriched GO and KEGG processes were performed with the significantly upregulated genes in endometriosis lesions, eutopic endometrium and normal endometrium. (D) Expression of selected pathway genes were shown for each cell of endometriosis, eutopic endometrium, or normal endometrium origin. Dot size corresponded to the percentage of cells in the cluster expressing a gene, and dot color corresponded to the average expression level for the gene in the cluster. Figure S4. DEG analysis compared cells from endometriosis lesions, eutopic endometrium, and normal endometrium within ECs and Eps by heatmap. Figure S5. Immunofluorescence in cells showed the C3, C7, StAR and S100A10 positive FBs in endometriosis lesions. Figure S6. (A) Contribution of each group to each cell state on Figure 2E. The majority of state 1 was occupied by FBs from the eutopic endometrium and normal endometrium. State 2 was primarily contained by ectopic and eutopic endometrium FBs, while state 3 contained FBs from all three groups. (B) Relative contributions of FBs from13 subclusters to each group. (C) Relative contributions of FBs from three groups to each cluster, as shown by the t-SNE plot. (D)Western blot analysis reflected si-StAR transfection efficiency. (E) Scratch wound healing determined the ability of migration between si-Ctrl and si- [file 13578_2021_637_MOESM1_ESM.zip › 13578_2021_637_MOESM10_ESM.tif]

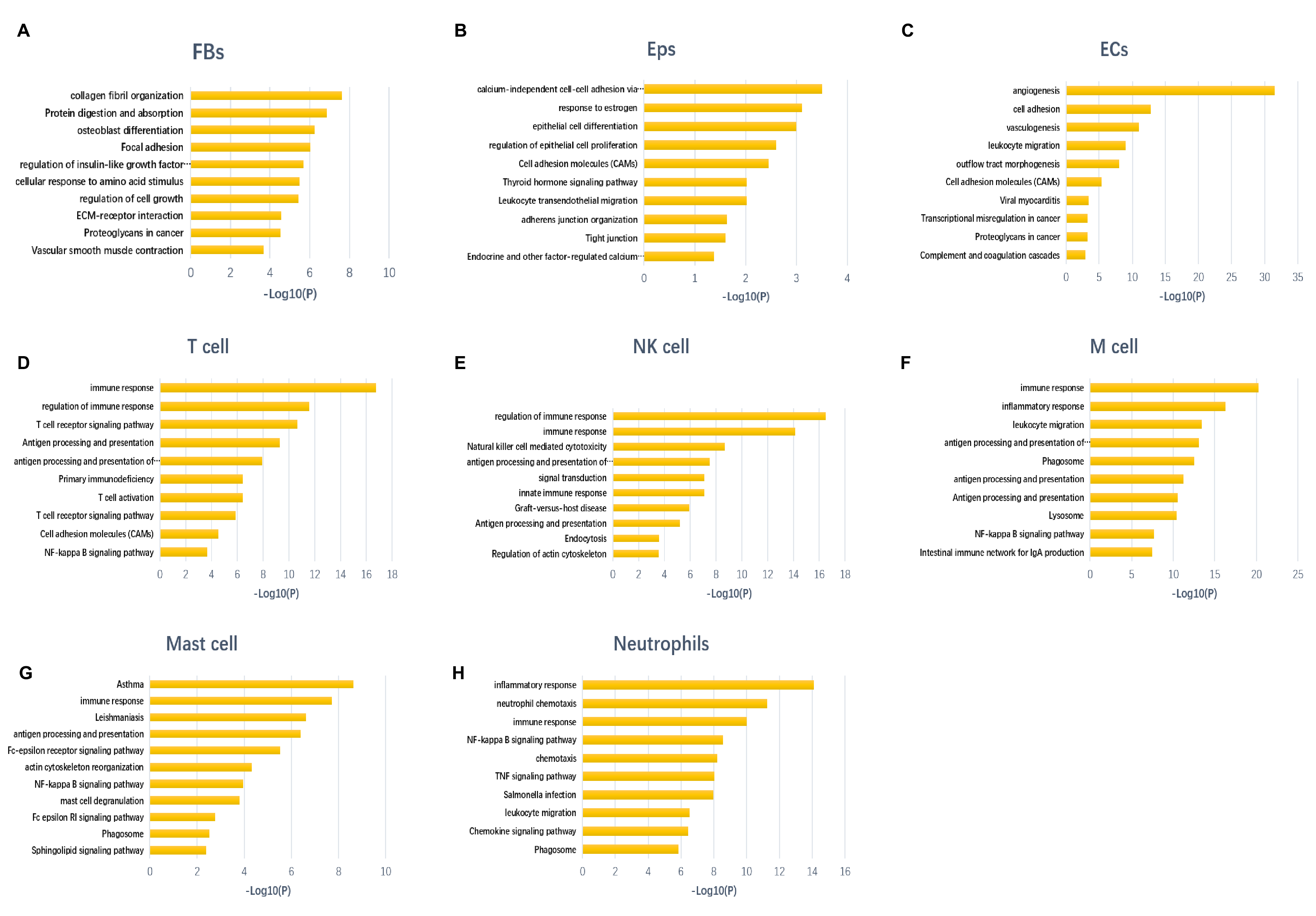

Supplement: Supplementary file 1 — Additional file 1: Figure S1. Study flow chart. Single-cell RNA-sequencing. Figure S2 a-g. Enriched functions of FBs, Eps, ECs, T cells, NK cells, M cells, ast cells and neutrophils by GO and KEGG analyses. Figure S3. Single-cell RNA-seq analysis revealed distinct characterization of cell populations in endometriosis lesions, eutopic endometrium and normal endometrium. (A) Similarity analyses of each cell population in endometriosis lesions, eutopic endometrium and normal endometrium. (B) Differential expression analysis was performed comparing whole cells from endometriosis lesions, eutopic endometrium and normal endometrium. (C) Representative significantly enriched GO and KEGG processes were performed with the significantly upregulated genes in endometriosis lesions, eutopic endometrium and normal endometrium. (D) Expression of selected pathway genes were shown for each cell of endometriosis, eutopic endometrium, or normal endometrium origin. Dot size corresponded to the percentage of cells in the cluster expressing a gene, and dot color corresponded to the average expression level for the gene in the cluster. Figure S4. DEG analysis compared cells from endometriosis lesions, eutopic endometrium, and normal endometrium within ECs and Eps by heatmap. Figure S5. Immunofluorescence in cells showed the C3, C7, StAR and S100A10 positive FBs in endometriosis lesions. Figure S6. (A) Contribution of each group to each cell state on Figure 2E. The majority of state 1 was occupied by FBs from the eutopic endometrium and normal endometrium. State 2 was primarily contained by ectopic and eutopic endometrium FBs, while state 3 contained FBs from all three groups. (B) Relative contributions of FBs from13 subclusters to each group. (C) Relative contributions of FBs from three groups to each cluster, as shown by the t-SNE plot. (D)Western blot analysis reflected si-StAR transfection efficiency. (E) Scratch wound healing determined the ability of migration between si-Ctrl and si- [file 13578_2021_637_MOESM1_ESM.zip › 13578_2021_637_MOESM2_ESM.tif]

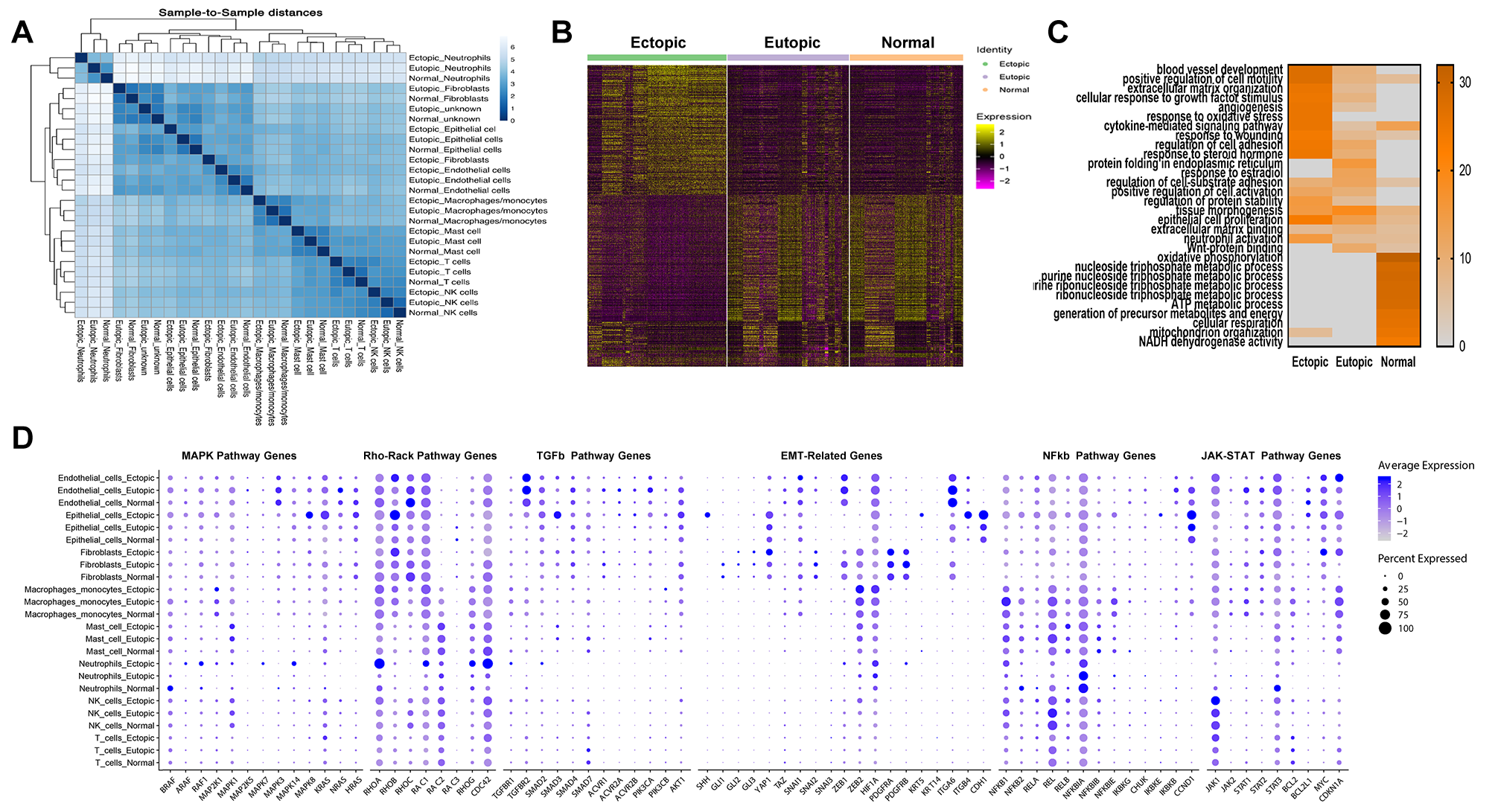

Supplement: Supplementary file 1 — Additional file 1: Figure S1. Study flow chart. Single-cell RNA-sequencing. Figure S2 a-g. Enriched functions of FBs, Eps, ECs, T cells, NK cells, M cells, ast cells and neutrophils by GO and KEGG analyses. Figure S3. Single-cell RNA-seq analysis revealed distinct characterization of cell populations in endometriosis lesions, eutopic endometrium and normal endometrium. (A) Similarity analyses of each cell population in endometriosis lesions, eutopic endometrium and normal endometrium. (B) Differential expression analysis was performed comparing whole cells from endometriosis lesions, eutopic endometrium and normal endometrium. (C) Representative significantly enriched GO and KEGG processes were performed with the significantly upregulated genes in endometriosis lesions, eutopic endometrium and normal endometrium. (D) Expression of selected pathway genes were shown for each cell of endometriosis, eutopic endometrium, or normal endometrium origin. Dot size corresponded to the percentage of cells in the cluster expressing a gene, and dot color corresponded to the average expression level for the gene in the cluster. Figure S4. DEG analysis compared cells from endometriosis lesions, eutopic endometrium, and normal endometrium within ECs and Eps by heatmap. Figure S5. Immunofluorescence in cells showed the C3, C7, StAR and S100A10 positive FBs in endometriosis lesions. Figure S6. (A) Contribution of each group to each cell state on Figure 2E. The majority of state 1 was occupied by FBs from the eutopic endometrium and normal endometrium. State 2 was primarily contained by ectopic and eutopic endometrium FBs, while state 3 contained FBs from all three groups. (B) Relative contributions of FBs from13 subclusters to each group. (C) Relative contributions of FBs from three groups to each cluster, as shown by the t-SNE plot. (D)Western blot analysis reflected si-StAR transfection efficiency. (E) Scratch wound healing determined the ability of migration between si-Ctrl and si- [file 13578_2021_637_MOESM1_ESM.zip › 13578_2021_637_MOESM3_ESM.tif]

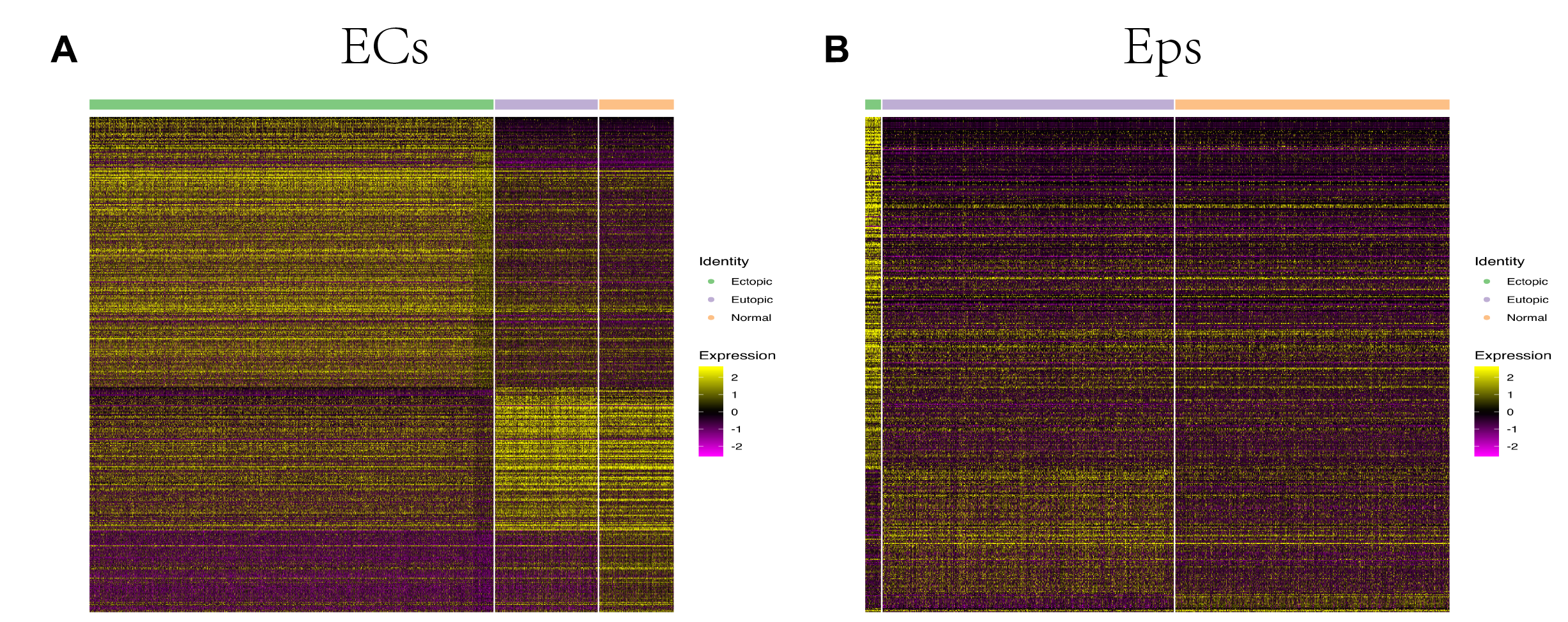

Supplement: Supplementary file 1 — Additional file 1: Figure S1. Study flow chart. Single-cell RNA-sequencing. Figure S2 a-g. Enriched functions of FBs, Eps, ECs, T cells, NK cells, M cells, ast cells and neutrophils by GO and KEGG analyses. Figure S3. Single-cell RNA-seq analysis revealed distinct characterization of cell populations in endometriosis lesions, eutopic endometrium and normal endometrium. (A) Similarity analyses of each cell population in endometriosis lesions, eutopic endometrium and normal endometrium. (B) Differential expression analysis was performed comparing whole cells from endometriosis lesions, eutopic endometrium and normal endometrium. (C) Representative significantly enriched GO and KEGG processes were performed with the significantly upregulated genes in endometriosis lesions, eutopic endometrium and normal endometrium. (D) Expression of selected pathway genes were shown for each cell of endometriosis, eutopic endometrium, or normal endometrium origin. Dot size corresponded to the percentage of cells in the cluster expressing a gene, and dot color corresponded to the average expression level for the gene in the cluster. Figure S4. DEG analysis compared cells from endometriosis lesions, eutopic endometrium, and normal endometrium within ECs and Eps by heatmap. Figure S5. Immunofluorescence in cells showed the C3, C7, StAR and S100A10 positive FBs in endometriosis lesions. Figure S6. (A) Contribution of each group to each cell state on Figure 2E. The majority of state 1 was occupied by FBs from the eutopic endometrium and normal endometrium. State 2 was primarily contained by ectopic and eutopic endometrium FBs, while state 3 contained FBs from all three groups. (B) Relative contributions of FBs from13 subclusters to each group. (C) Relative contributions of FBs from three groups to each cluster, as shown by the t-SNE plot. (D)Western blot analysis reflected si-StAR transfection efficiency. (E) Scratch wound healing determined the ability of migration between si-Ctrl and si- [file 13578_2021_637_MOESM1_ESM.zip › 13578_2021_637_MOESM4_ESM.tif]

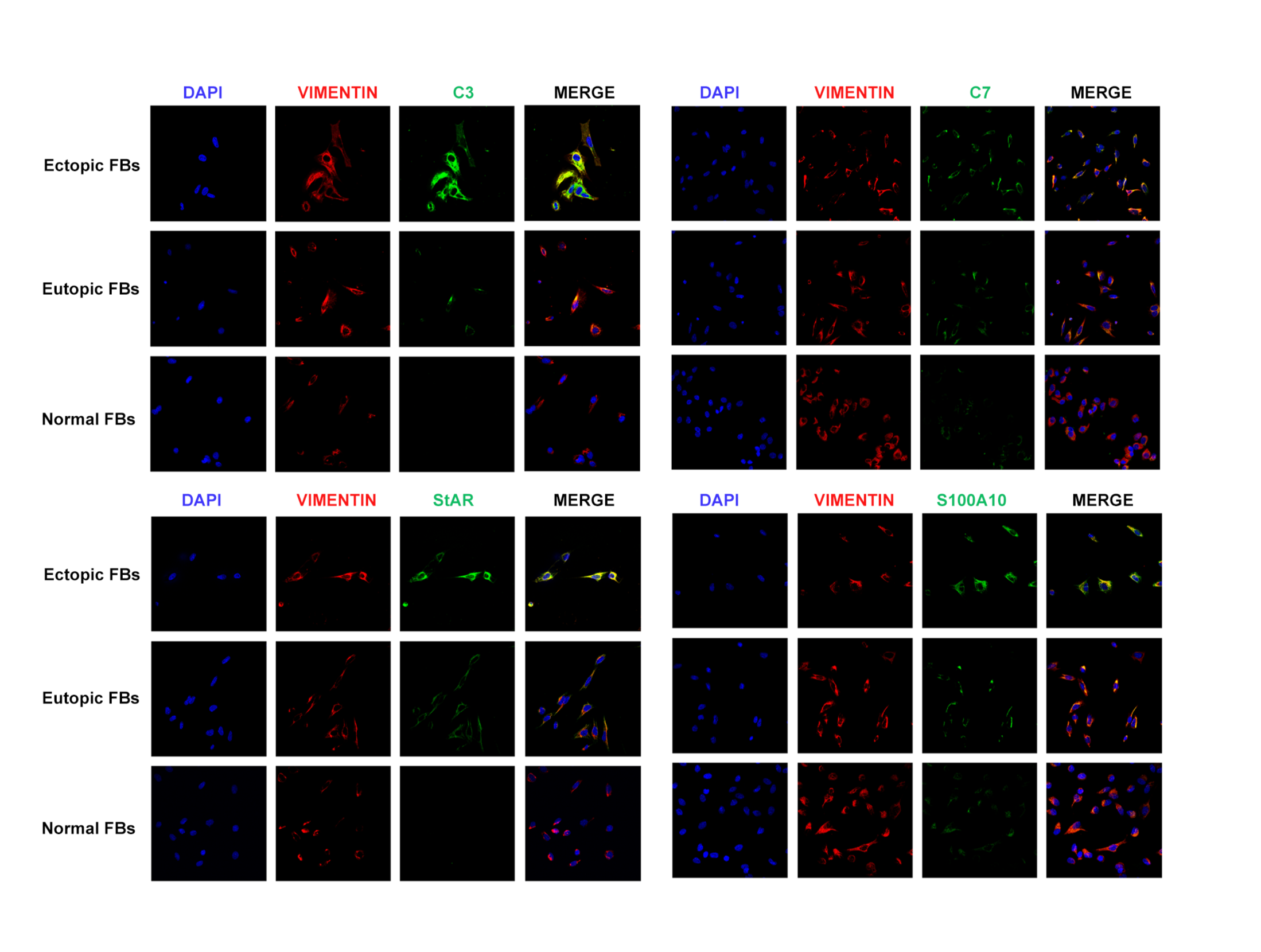

Supplement: Supplementary file 1 — Additional file 1: Figure S1. Study flow chart. Single-cell RNA-sequencing. Figure S2 a-g. Enriched functions of FBs, Eps, ECs, T cells, NK cells, M cells, ast cells and neutrophils by GO and KEGG analyses. Figure S3. Single-cell RNA-seq analysis revealed distinct characterization of cell populations in endometriosis lesions, eutopic endometrium and normal endometrium. (A) Similarity analyses of each cell population in endometriosis lesions, eutopic endometrium and normal endometrium. (B) Differential expression analysis was performed comparing whole cells from endometriosis lesions, eutopic endometrium and normal endometrium. (C) Representative significantly enriched GO and KEGG processes were performed with the significantly upregulated genes in endometriosis lesions, eutopic endometrium and normal endometrium. (D) Expression of selected pathway genes were shown for each cell of endometriosis, eutopic endometrium, or normal endometrium origin. Dot size corresponded to the percentage of cells in the cluster expressing a gene, and dot color corresponded to the average expression level for the gene in the cluster. Figure S4. DEG analysis compared cells from endometriosis lesions, eutopic endometrium, and normal endometrium within ECs and Eps by heatmap. Figure S5. Immunofluorescence in cells showed the C3, C7, StAR and S100A10 positive FBs in endometriosis lesions. Figure S6. (A) Contribution of each group to each cell state on Figure 2E. The majority of state 1 was occupied by FBs from the eutopic endometrium and normal endometrium. State 2 was primarily contained by ectopic and eutopic endometrium FBs, while state 3 contained FBs from all three groups. (B) Relative contributions of FBs from13 subclusters to each group. (C) Relative contributions of FBs from three groups to each cluster, as shown by the t-SNE plot. (D)Western blot analysis reflected si-StAR transfection efficiency. (E) Scratch wound healing determined the ability of migration between si-Ctrl and si- [file 13578_2021_637_MOESM1_ESM.zip › 13578_2021_637_MOESM5_ESM.tif]

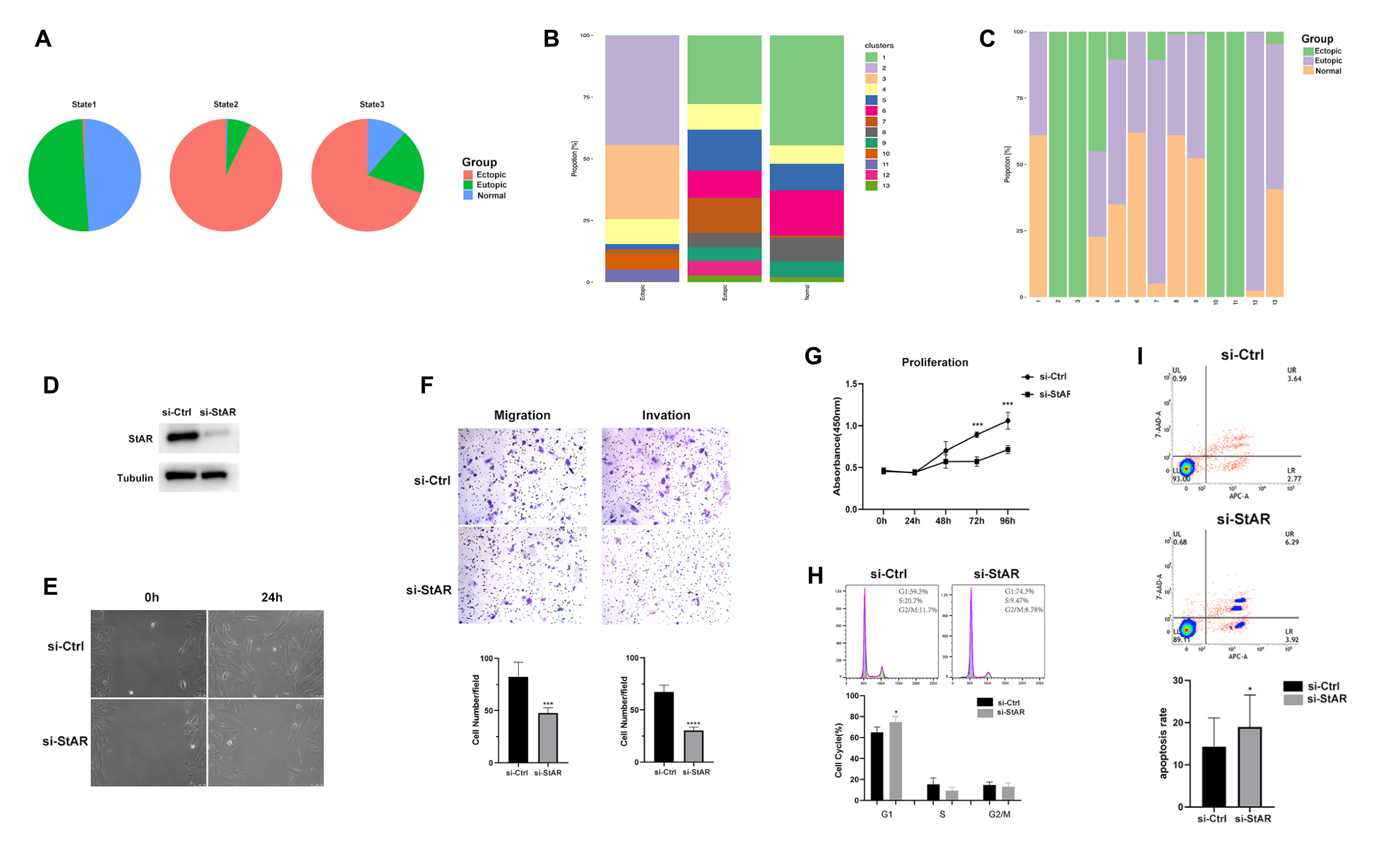

Supplement: Supplementary file 1 — Additional file 1: Figure S1. Study flow chart. Single-cell RNA-sequencing. Figure S2 a-g. Enriched functions of FBs, Eps, ECs, T cells, NK cells, M cells, ast cells and neutrophils by GO and KEGG analyses. Figure S3. Single-cell RNA-seq analysis revealed distinct characterization of cell populations in endometriosis lesions, eutopic endometrium and normal endometrium. (A) Similarity analyses of each cell population in endometriosis lesions, eutopic endometrium and normal endometrium. (B) Differential expression analysis was performed comparing whole cells from endometriosis lesions, eutopic endometrium and normal endometrium. (C) Representative significantly enriched GO and KEGG processes were performed with the significantly upregulated genes in endometriosis lesions, eutopic endometrium and normal endometrium. (D) Expression of selected pathway genes were shown for each cell of endometriosis, eutopic endometrium, or normal endometrium origin. Dot size corresponded to the percentage of cells in the cluster expressing a gene, and dot color corresponded to the average expression level for the gene in the cluster. Figure S4. DEG analysis compared cells from endometriosis lesions, eutopic endometrium, and normal endometrium within ECs and Eps by heatmap. Figure S5. Immunofluorescence in cells showed the C3, C7, StAR and S100A10 positive FBs in endometriosis lesions. Figure S6. (A) Contribution of each group to each cell state on Figure 2E. The majority of state 1 was occupied by FBs from the eutopic endometrium and normal endometrium. State 2 was primarily contained by ectopic and eutopic endometrium FBs, while state 3 contained FBs from all three groups. (B) Relative contributions of FBs from13 subclusters to each group. (C) Relative contributions of FBs from three groups to each cluster, as shown by the t-SNE plot. (D)Western blot analysis reflected si-StAR transfection efficiency. (E) Scratch wound healing determined the ability of migration between si-Ctrl and si- [file 13578_2021_637_MOESM1_ESM.zip › 13578_2021_637_MOESM6_ESM.tif]

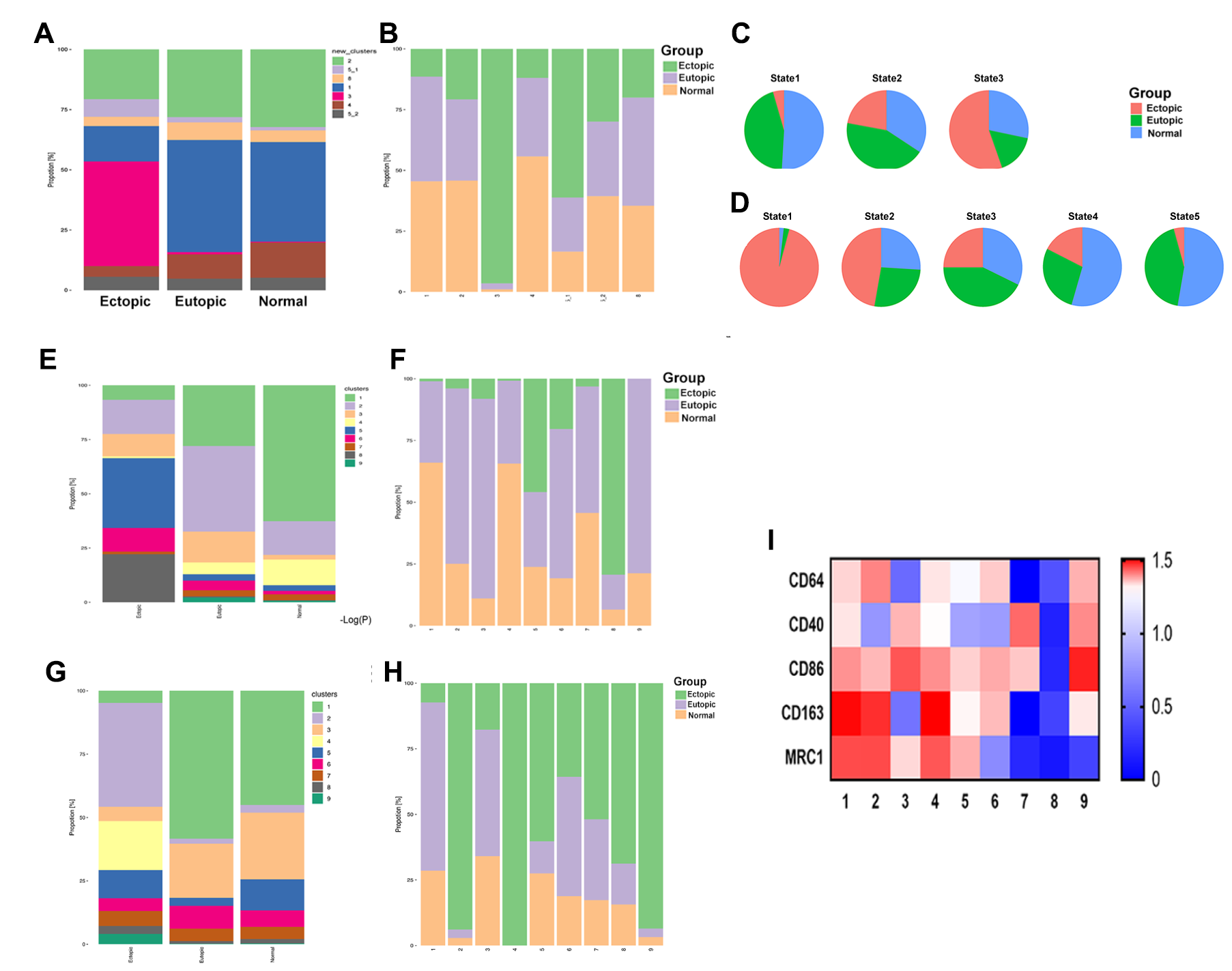

Supplement: Supplementary file 1 — Additional file 1: Figure S1. Study flow chart. Single-cell RNA-sequencing. Figure S2 a-g. Enriched functions of FBs, Eps, ECs, T cells, NK cells, M cells, ast cells and neutrophils by GO and KEGG analyses. Figure S3. Single-cell RNA-seq analysis revealed distinct characterization of cell populations in endometriosis lesions, eutopic endometrium and normal endometrium. (A) Similarity analyses of each cell population in endometriosis lesions, eutopic endometrium and normal endometrium. (B) Differential expression analysis was performed comparing whole cells from endometriosis lesions, eutopic endometrium and normal endometrium. (C) Representative significantly enriched GO and KEGG processes were performed with the significantly upregulated genes in endometriosis lesions, eutopic endometrium and normal endometrium. (D) Expression of selected pathway genes were shown for each cell of endometriosis, eutopic endometrium, or normal endometrium origin. Dot size corresponded to the percentage of cells in the cluster expressing a gene, and dot color corresponded to the average expression level for the gene in the cluster. Figure S4. DEG analysis compared cells from endometriosis lesions, eutopic endometrium, and normal endometrium within ECs and Eps by heatmap. Figure S5. Immunofluorescence in cells showed the C3, C7, StAR and S100A10 positive FBs in endometriosis lesions. Figure S6. (A) Contribution of each group to each cell state on Figure 2E. The majority of state 1 was occupied by FBs from the eutopic endometrium and normal endometrium. State 2 was primarily contained by ectopic and eutopic endometrium FBs, while state 3 contained FBs from all three groups. (B) Relative contributions of FBs from13 subclusters to each group. (C) Relative contributions of FBs from three groups to each cluster, as shown by the t-SNE plot. (D)Western blot analysis reflected si-StAR transfection efficiency. (E) Scratch wound healing determined the ability of migration between si-Ctrl and si- [file 13578_2021_637_MOESM1_ESM.zip › 13578_2021_637_MOESM7_ESM.tif]

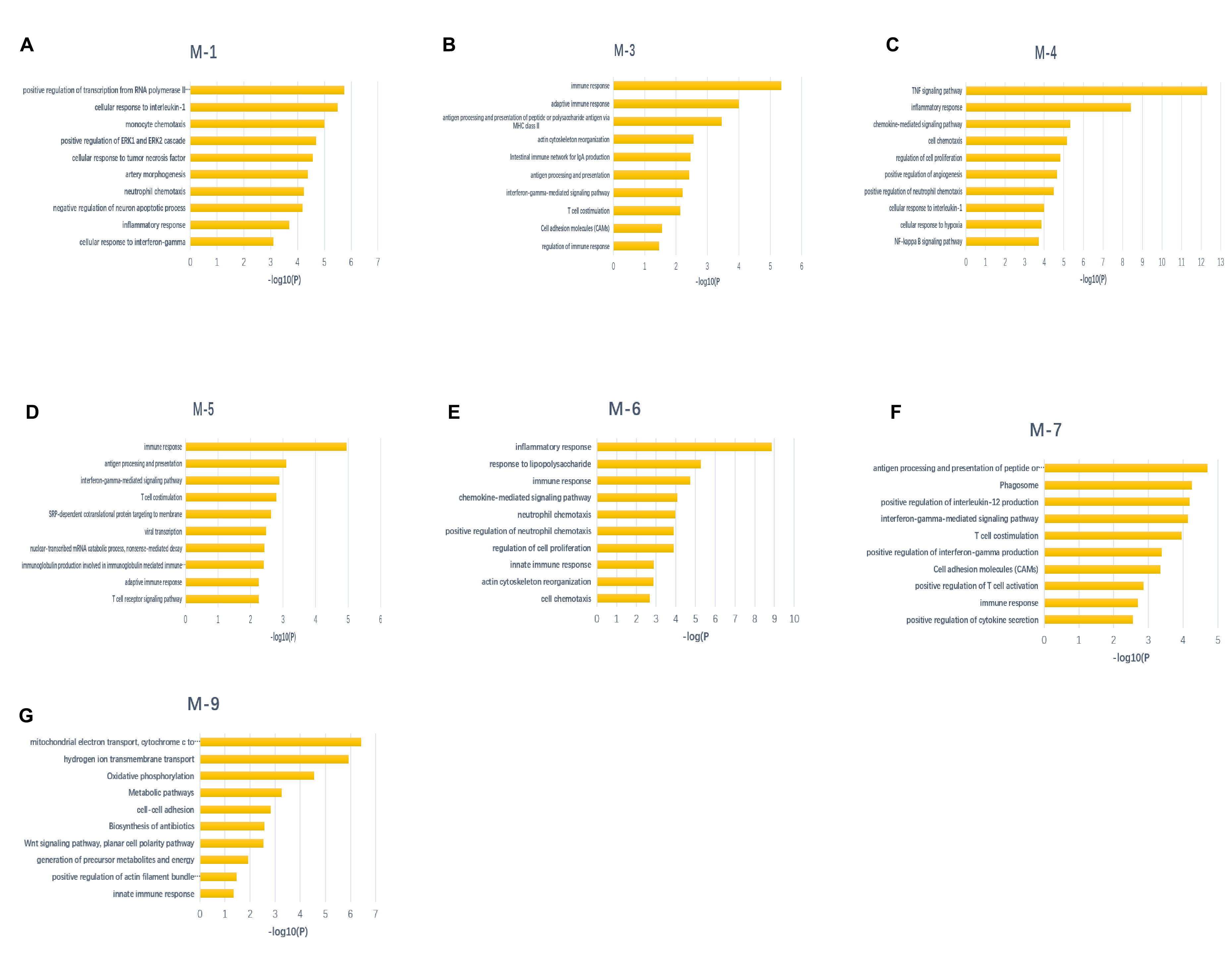

Supplement: Supplementary file 1 — Additional file 1: Figure S1. Study flow chart. Single-cell RNA-sequencing. Figure S2 a-g. Enriched functions of FBs, Eps, ECs, T cells, NK cells, M cells, ast cells and neutrophils by GO and KEGG analyses. Figure S3. Single-cell RNA-seq analysis revealed distinct characterization of cell populations in endometriosis lesions, eutopic endometrium and normal endometrium. (A) Similarity analyses of each cell population in endometriosis lesions, eutopic endometrium and normal endometrium. (B) Differential expression analysis was performed comparing whole cells from endometriosis lesions, eutopic endometrium and normal endometrium. (C) Representative significantly enriched GO and KEGG processes were performed with the significantly upregulated genes in endometriosis lesions, eutopic endometrium and normal endometrium. (D) Expression of selected pathway genes were shown for each cell of endometriosis, eutopic endometrium, or normal endometrium origin. Dot size corresponded to the percentage of cells in the cluster expressing a gene, and dot color corresponded to the average expression level for the gene in the cluster. Figure S4. DEG analysis compared cells from endometriosis lesions, eutopic endometrium, and normal endometrium within ECs and Eps by heatmap. Figure S5. Immunofluorescence in cells showed the C3, C7, StAR and S100A10 positive FBs in endometriosis lesions. Figure S6. (A) Contribution of each group to each cell state on Figure 2E. The majority of state 1 was occupied by FBs from the eutopic endometrium and normal endometrium. State 2 was primarily contained by ectopic and eutopic endometrium FBs, while state 3 contained FBs from all three groups. (B) Relative contributions of FBs from13 subclusters to each group. (C) Relative contributions of FBs from three groups to each cluster, as shown by the t-SNE plot. (D)Western blot analysis reflected si-StAR transfection efficiency. (E) Scratch wound healing determined the ability of migration between si-Ctrl and si- [file 13578_2021_637_MOESM1_ESM.zip › 13578_2021_637_MOESM8_ESM.tif]

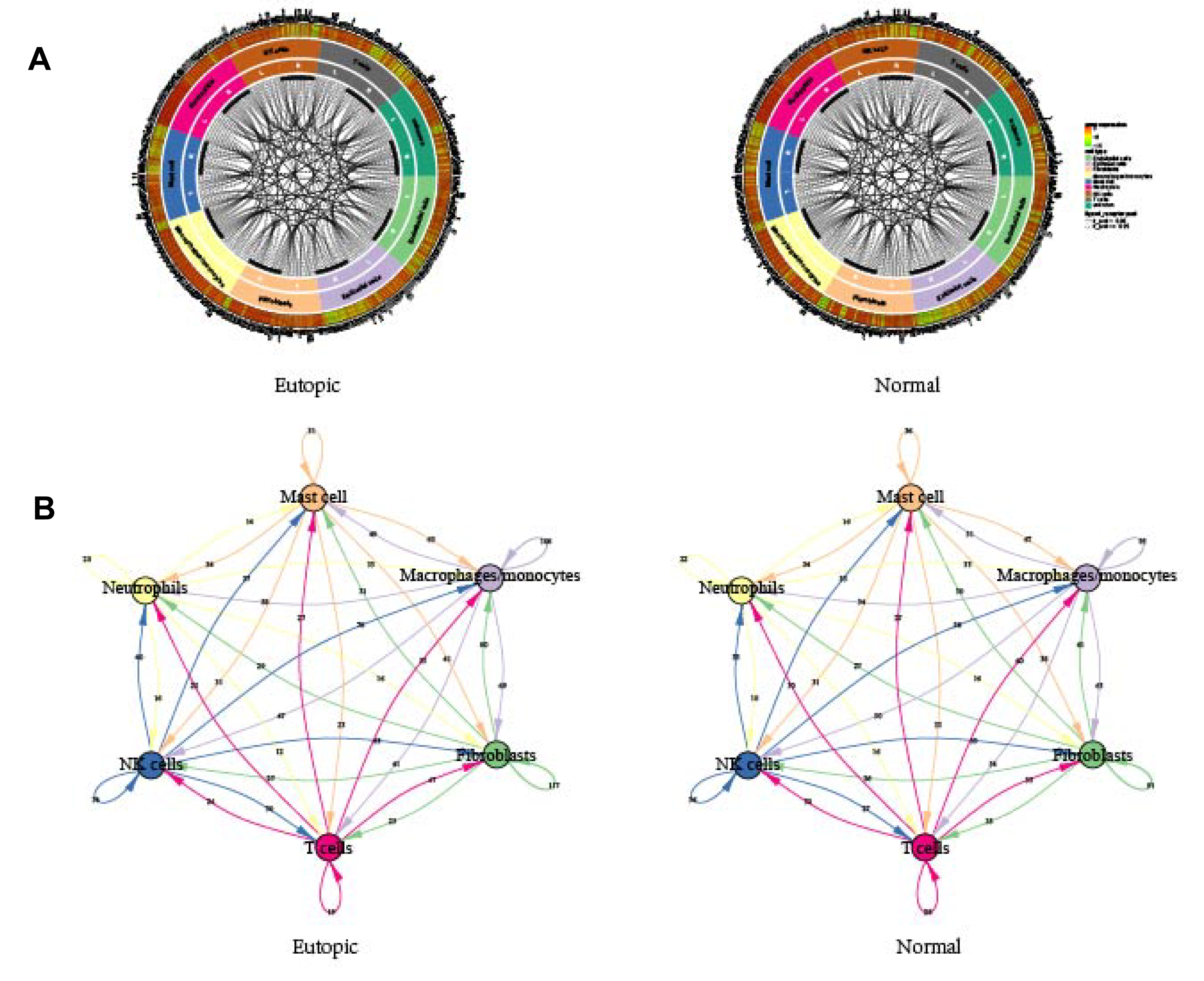

Supplement: Supplementary file 1 — Additional file 1: Figure S1. Study flow chart. Single-cell RNA-sequencing. Figure S2 a-g. Enriched functions of FBs, Eps, ECs, T cells, NK cells, M cells, ast cells and neutrophils by GO and KEGG analyses. Figure S3. Single-cell RNA-seq analysis revealed distinct characterization of cell populations in endometriosis lesions, eutopic endometrium and normal endometrium. (A) Similarity analyses of each cell population in endometriosis lesions, eutopic endometrium and normal endometrium. (B) Differential expression analysis was performed comparing whole cells from endometriosis lesions, eutopic endometrium and normal endometrium. (C) Representative significantly enriched GO and KEGG processes were performed with the significantly upregulated genes in endometriosis lesions, eutopic endometrium and normal endometrium. (D) Expression of selected pathway genes were shown for each cell of endometriosis, eutopic endometrium, or normal endometrium origin. Dot size corresponded to the percentage of cells in the cluster expressing a gene, and dot color corresponded to the average expression level for the gene in the cluster. Figure S4. DEG analysis compared cells from endometriosis lesions, eutopic endometrium, and normal endometrium within ECs and Eps by heatmap. Figure S5. Immunofluorescence in cells showed the C3, C7, StAR and S100A10 positive FBs in endometriosis lesions. Figure S6. (A) Contribution of each group to each cell state on Figure 2E. The majority of state 1 was occupied by FBs from the eutopic endometrium and normal endometrium. State 2 was primarily contained by ectopic and eutopic endometrium FBs, while state 3 contained FBs from all three groups. (B) Relative contributions of FBs from13 subclusters to each group. (C) Relative contributions of FBs from three groups to each cluster, as shown by the t-SNE plot. (D)Western blot analysis reflected si-StAR transfection efficiency. (E) Scratch wound healing determined the ability of migration between si-Ctrl and si- [file 13578_2021_637_MOESM1_ESM.zip › 13578_2021_637_MOESM9_ESM.tif]
