# Supplementary material for: Single-cell transcriptomic analysis of endometriosis provides insights into fibroblast fates and immune cell heterogeneity
Source: Cell Biosci. 2021 Jul 7;11:125. doi: 10.1186/s13578-021-00637-x (PMC8261960; doi:10.1186/s13578-021-00637-x)
Supplement: Supplementary file 2 — Additional file 2. Characteristics of samples included in this study. [file 13578_2021_637_MOESM2_ESM.docx]

**Table S1. Sequencing statistics for the three endometriosis samples, three eutopic endometrium samples and three normal endometrium sample included in this study**

| **Patient ID** | **Sample type** | **10X version*** | **UMIs** | **Cells** | **UMIs/cell** | **Mean reads per cell** | **Median genes per cell** |
| --- | --- | --- | --- | --- | --- | --- | --- |
| Patient 1 | Endometriosis cyst | V3 | 558776785 | 7980 | 6949 | 70022 | 2486 |
| Patient 2 | Endometriosis cyst | V3 | 503758754 | 9485 | 8310 | 53111 | 2125 |
| Patient 3 | Endometriosis cyst | V3 | 608404695 | 5907 | 10070 | 102997 | 2763 |
| Patient 1 | Eutopic endometrium | V3 | 534976964 | 6466 | 6243 | 82737 | 2225 |
| Patient 2 | Eutopic endometrium | V3 | 551013726 | 8562 | 5508 | 64356 | 1918 |
| Patient 3 | Eutopic endometrium | V3 | 576475628 | 6872 | 9612 | 83888 | 2882 |
| Patient 4 | Normal endometrium | V3 | 578230184 | 5904 | 5583 | 97939 | 1861 |
| Patient 5 | Normal endometrium | V3 | 564406720 | 7089 | 5532 | 79617 | 1762 |
| Patient 6 | Normal endometrium | V3 | 611919187 | 8838 | 12410 | 69237 | 3199 |

*Single-cell suspensions were converted into single-cell RNA-seq libraries using a commercially available DROP-seq protocol (10X genomics) following version 3 chemistry.

UMI, unique molecular identifier, equivalent to a unique detected transcript.
